# GM2 Gangliosidoses: Clinical Features, Pathophysiological Aspects, and Current Therapies

**DOI:** 10.3390/ijms21176213

**Published:** 2020-08-27

**Authors:** Andrés Felipe Leal, Eliana Benincore-Flórez, Daniela Solano-Galarza, Rafael Guillermo Garzón Jaramillo, Olga Yaneth Echeverri-Peña, Diego A. Suarez, Carlos Javier Alméciga-Díaz, Angela Johana Espejo-Mojica

**Affiliations:** 1 Institute for the Study of Inborn Errors of Metabolism, Faculty of Science, Pontificia Universidad Javeriana, Bogotá 110231, Colombia; lealb.af@javeriana.edu.co (A.F.L.); elianabenincore@javeriana.edu.co (E.B.-F); aura.solano@javeriana.edu.co (D.S.-G.); rafael.garzon@javeriana.edu.co (R.G.G.J.); oyecheve@javeriana.edu.co (O.Y.E.-P.); suarezd.i@javeriana.edu.co (D.A.S.); 2 Faculty of Medicine, Universidad Nacional de Colombia, Bogotá 110231, Colombia

**Keywords:** lysosomal storage disorders, GM2 gangliosidoses, Tay–Sachs disease, Sandhoff disease, β-Hexosaminidases, therapeutic alternatives

## Abstract

GM2 gangliosidoses are a group of pathologies characterized by GM2 ganglioside accumulation into the lysosome due to mutations on the genes encoding for the β-hexosaminidases subunits or the GM2 activator protein. Three GM2 gangliosidoses have been described: Tay–Sachs disease, Sandhoff disease, and the AB variant. Central nervous system dysfunction is the main characteristic of GM2 gangliosidoses patients that include neurodevelopment alterations, neuroinflammation, and neuronal apoptosis. Currently, there is not approved therapy for GM2 gangliosidoses, but different therapeutic strategies have been studied including hematopoietic stem cell transplantation, enzyme replacement therapy, substrate reduction therapy, pharmacological chaperones, and gene therapy. The blood–brain barrier represents a challenge for the development of therapeutic agents for these disorders. In this sense, alternative routes of administration (e.g., intrathecal or intracerebroventricular) have been evaluated, as well as the design of fusion peptides that allow the protein transport from the brain capillaries to the central nervous system. In this review, we outline the current knowledge about clinical and physiopathological findings of GM2 gangliosidoses, as well as the ongoing proposals to overcome some limitations of the traditional alternatives by using novel strategies such as molecular Trojan horses or advanced tools of genome editing.

## 1. Introduction

Gangliosides are a group of glycosphingolipids mainly located in the neuronal cell membrane that are responsible for several pivotal biological functions for the correct functioning of the central nervous system (CNS) [1]. About 5% of all brain gangliosides correspond to GM2 gangliosides [2,3]. In normal conditions, GM2 gangliosides are catabolized by the lysosomal hydrolases β-hexosaminidases (Hex, EC 3.2.1.52) through the hydrolysis of the N-acetylgalactosamine residues [4]. Hex are a subset of isozymes formed by the dimerization of α and β subunits: HexA (αβ), HexB (ββ) and HexS (αα). In addition, GM2 gangliosides degradation involves the GM2 activator protein (GM2-AP), which present the gangliosides to α subunit of HexA [1]. Mutations in the genes encoding for α (*HEXA*), β (*HEXB*) or GM2-AP (*GM2A*) proteins affect the lysosomal degradation of GM2 ganglioside and other glycolipids, causing their accumulation into the lysosome and the GM2 gangliosidoses Tay–Sachs (TSD, OMIM #272800), Sandhoff (SD, OMIM #268800), or GM2-activator protein deficiency (AB variant; OMIM #272750), respectively [4]. Upon the GM2 ganglioside accumulation, several cytotoxic effects take place mainly in neurons, which frequently cause neuronal death [5]. Individuals with GM2 gangliosidoses have a progressive neurological impairment including motor deficits, progressive weakness, hypotonia, decreased responsiveness, vision deterioration, and seizures [6]. Unlike TSD patients, SD patients may present systemic manifestations as organomegalies [7,8]. The diagnosis for these disorders begins with recognition of the clinical characteristics, which is follows by the measurement of the enzymatic activity that can be confirmed by mutation analysis [9,10].

Both the understanding of physiopathology and the development of therapies for GM2 gangliosidoses have benefited from the different animal models available for these disorders. These animal models include mice, cats, and sheep, which mimics some of the biochemical and physiological characteristics of GM2 gangliosidoses [11]. An ideal TSD mouse model was recently developed through a combined deficiency of HexA and Neu3, which mimics the neuropathological and clinical abnormalities of classical early-onset TSD patients and may provide a valuable tool for treatments development for this condition [12].

Several therapeutic approaches have been evaluated for GM2 gangliosidoses, including enzyme replacement therapy, hematopoietic stem cell transplantation, pharmacological chaperones, substrate reduction therapy, and gene therapy. Nevertheless, currently there is not an approved therapy for these disorders. The efficacy of the therapeutic approaches is affected, among others things, by the blood–brain barrier (BBB) that limits the access of intravenous therapeutic agents to the CNS [13]. In this sense, strategies involving chimeric recombinant enzymes, direct brain injection, or the development of vehicles to target proteins to the brain have shown promising advantages respect to conventional administration strategies [14]. Likewise, the development of novel gene editing tools, as clustered regularly interspaced short palindromic repeats/CRISPR-associated protein 9 (CRISPR/Cas9), have supposed a new horizon to the treatment of the lysosomal storage disorders including the GM2 gangliosidoses [15]. In this paper, we provide a critical review about physiopathology features and diagnosis of these diseases as well as a major up to date about the development of therapies for GM2 gangliosidoses.

## 2. Gangliosides: Structure and Physiological Role

Gangliosides are complex glycolipids composed of a ceramide linked to a glycan with at least one sialic acid [2]. Currently, over 180 gangliosides have been identified in vertebrates [2,16]. In humans, GM3 ganglioside is predominantly in peripheral tissues such as liver, adipose tissue, aorta, and platelets [17]; whereas GM1, GD1a, GD1b, GT1b, and GQ1b are the major gangliosides in human brain (~95%) [18]. The remaining 5% of brain gangliosides corresponds to other gangliosides among which GM2 is found [2,3,19]. Gangliosides are distributed in caveolae-rich microdomains of the plasma membrane [16,20,21], where they perform crucial functions such as membrane organization [21], neuronal differentiation [20,22], cell adhesion [23], signal transduction [24], inflammation [3], and neurite outgrowth [22,25], among others.

The *novo* biosynthesis of gangliosides starts with the formation of ceramide in the cytoplasmic side of rough endoplasmic reticulum (RER) and that ends in the *trans*-Golgi network through the sequential addition of carbohydrates in a process catalyzed by several glycosyltransferases to generate lactosylceramide [1,26]. Subsequently, the LacCer-α-2–3 sialyltransferase adds sialic acid to form GM3 gangliosides and other precursor gangliosides [26]. This GM3 ganglioside acts as precursor for the synthesis of more complex gangliosides such as GM2, by the action of β-1,4-N-acetylgalactosaminyl transferase (GM2/GD2 synthase), which transfers the N-acetylgalactosamine residue to the GM3 structure [4,26,27]. GM1 and GD1a gangliosides differ from GM2 by the number and type of monosaccharides presents, as well as by the number of sialic acid residues.

## 3. β-Hexosaminidases: Synthesis, Transport, and Catalytic Functions

β-hexosaminidases are dimeric lysosomal enzymes composed by α and/or β subunits to form HexA (αβ), HexB (ββ), and HexS (αα) isoforms [4]. Genes of α (*HEXA*) and β (*HEXB*) subunits are located in chromosome 15q23 and 5q13.3, respectively [4]. Fourteen exons and 13 introns are described for both genes which share a 60% of identity, suggesting a common ancestor [28]. Early studies using pulse and chase analysis showed that Hex are synthesized as long precursors of 67 (α) and 63 (β) kDa that are proteolytic processed to 54 and 52 kDa peptides, respectively [29,30]. The β subunit suffers further proteolysis to obtain a mature form of 29 kDa and other smaller peptides that remain linked by disulfide bonds [29]. Two major cleavages points for α precursors have been identified: (1) alanine 22 that allows removing the signal peptide (22 a.a.) into the ER and (2) lysine 86 that is followed by lysosomal exopeptidase-mediated trimming of three amino acids to give the mature form of α subunit into the lysosome [28,31]. For β precursors, cleavages points are found in valine 42 that removes signal peptide (42 a.a.) and alanine 45 [32]. Furthermore, the mature subunit is nicked internally in the valine 48, threonine 122, and lysine 315, which remain joined through disulfide bonds [32].

Post-translational modifications of Hex, as N-glycosylations and phosphorylations, are carried out during the ER-Golgi traffic of the proteins [33]. For α chains, asparagine (Asn) 115, 157, and 295 have been identified as putative N-glycosylation sites [34]; whereas Asn 84, 142, 190, and 327 have been described for β chains [34,35]. Additionally, early studies suggested that terminal mannoses present on Asn84, Asn115, and Asn295 N-glycosylations must be phosphorylated to be recognized by the mannose-6 phosphate receptor (M6PR) [33]. After these modifications take place, the subunits are dimerized to obtain the active enzymes [33]. Although it is not clear the organelle in which Hex are dimerized; some authors suggest that this process is carried out into the *trans*-Golgi network (TGN) before being targeted to the endosome-lysosome pathway [36,37,38]. These enzymes can also reach the extracellular compartment through sorting from TGN, which can be then taken up by neighboring or secreting cells through fluid-membrane endocytosis [4]. The uptake of these enzymes by neighboring cells constitutes the cross-correction mechanism that is the base of the main therapeutic strategies for lysosomal storage diseases (LSDs). In addition, the HexA has also been identified on the plasma membrane of in vitro cultured fibroblasts, which has shown activity against the GM2 ganglioside [39]. However, the in vivo physiological role of this membrane-bound enzyme and the transport mechanism from TGN it is not completely understood yet.

**Figure 1:**
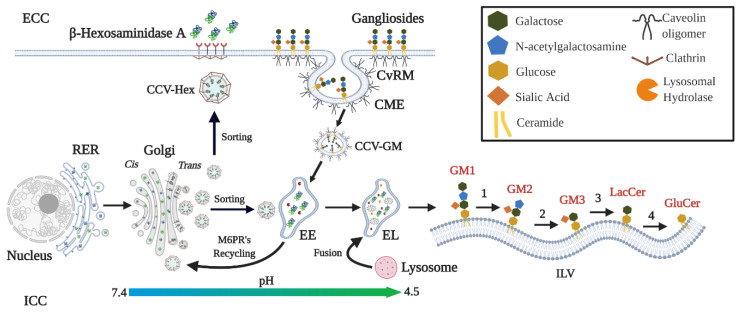
M6PR-dependent transport of β-Hexosaminidase A and ganglioside degradation. α and β subunits of Hex are synthesized in the rough endoplasmic reticulum (RER) and transported to the *Cis*-Golgi network. In this compartment, Hex is subject to N-glycosylations and phosphorylations from *Cis*-Golgi network to the *Trans*-Golgi network [33]. Monomers are dimerized in the *Trans*-Golgi network and coupled to mannose-6 phosphate receptors (M6PR) [33,37]. New vesicles are sorted to both early endosomes (EE) and to the secretory pathway, where can be uptake by neighbor cells through M6PR [37,45]. Hexosaminidases are dissociated from the M6PR in the EE; which allows the M6PR recycling to the *Trans*-Golgi network by both clathrin-dependent and independent mechanisms [37]. On the other side, gangliosides are placed in caveolae-rich microdomains (CvRM), and in the turnover of the plasma membrane undergo caveolae-mediated endocytosis (CME) [1,38]. New caveosomes containing gangliosides (CCV-GM) reach the EE and further fusion events result in a late endosome, which can be fused with the lysosome to give rise to the endo-lysosome (EL, pH: 4.5) [37]. Gangliosides degradation starts with the hydrolysis of the galactose of the GM1 ganglioside to generate GM2 ganglioside which are harbored on intralysosomal vesicles (ILV) [1]. GM2 interacts with HexA through a GM2AP-mediated mechanism to removes the N-acetylgalactosamine resides [38]. Additional reactions implied in the ganglioside degradation to glucosylceramide (GluCer) are shown. The enzymes of each reaction are as follow: 1 and 4: β-Galactosidase/GM2AP, 2: β-Hexosaminidase A/GM2AP, and 3: Neuraminidase. LacCer: Lactoceramide. ECC: Extracellular compartment. ICC: Intracellular compartment.

**Figure 2:**
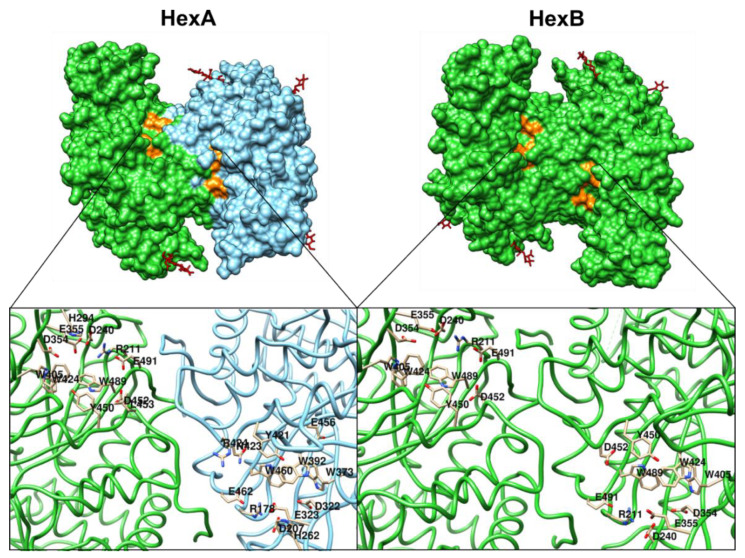
Structure of HexA and HexB. HexA (PDB 2gjx) isolated from human placenta, while HexB (PDB 1o7a) was recombinantly expressed in insect cells. α- and β-subunits are colored in light blue and green, respectively. N-glycans and active sites are colored in red and orange, respectively. The residues present in the active site of each subunit are also shown.

Substrate degradation requires the dimerization of HexA and HexB. Noteworthy, only HexA can hydrolyze the N-acetylgalactosamine present in GM2 ganglioside; while both HexA and HexB hydrolyze a variety of substrates including glycosaminoglycans, glycolipids, and glycoproteins [4,33,40,41]. In addition, the hydrolysis of galactose in the GM1 ganglioside structure is necessary for the catalytic activity of the HexA on the GM2 ganglioside (Figure 1) [38]. For the HexA, two active sites have been described, one on each α and β subunits (Figure 2) [33,42]. Glutamate 323 and 355 in the α and β subunits, respectively, act as general residues that allow the protonation of the glycosidic oxygen atom; whereas aspartate 322 and 354 in the α and β subunit, respectively, contribute to the necessary stabilization during the nucleophilic attack to the N-acetylgalactosamine [33,42]. In addition, it has been proposed that arginine 424 of the α subunit form hydrogen bonds with the carboxylate group of the sialic acid; whereas aspartate 452 in the β subunit would repeal the sialic acid binding [33,42]. This fact may explain the differences in the affinities of the natural and artificial substrates for HexA and HexB [40]. In contrast to globoside degradation by HexB, the GM2 ganglioside degradation requires the GM2-AP mediation [43]. GM2-AP is considered to be a lipid transporter protein that removes the GM2 ganglioside from the endosome membranes derived from the plasma membrane internalized during caveolae-mediated endocytosis [1]. In vitro approaches have predicted that GM2-AP simultaneous interactions between the GM2 ganglioside and the α subunit of HexA are necessary for the hydrolysis of N-acetylgalactosamine residue [1,44].

## 4. Mutations of β-Hexosaminidases A and B, and GM2 Activator Protein

**Figure 3:**
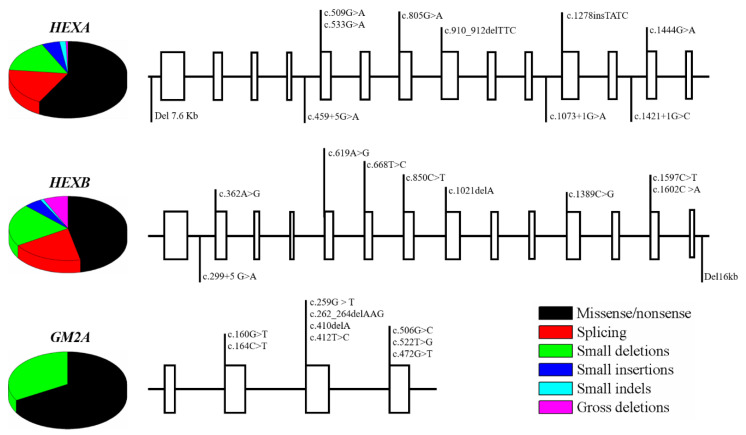
Common mutations on *HEXA*, *HEXB*, and *GM2A genes***.** The figure shows some of the most common mutations identified on *HEXA*, *HEXB*, and *GM2A*, as well as their distribution throughout the gene. Mutations can be found either on exons (boxes), introns, and the 5′ and 3′UTRs. 14 exons and 13 introns are represented to *HEXA* and *HEXB*, whereas 4 exons and 3 introns are shown for *GM2A*. This figure was made according to the reviewed in [49,50,51,52,53,54,55].

According to *The Human Gene Mutation Database*, currently 181, 103, and 9 mutations have been reported for *HEXA*, *HEXB*, and *GM2A* genes, respectively, including missense/nonsense, splicing, small deletion and indels, and gross deletions (Figure 3) [46,47,48]. Although the type and frequency of mutations have been linked to the demographic origin of the patients, for *HEXA* the most representative mutation is the transition c.533 G > A that changes arginine by histidine (p.R178H) and affects the catalytic site of the α subunit, altering its function and stability [46,47,48,49]. In the case of *HEXB*; a frequent mutation is c.445 + 1 G > A, which occurs in a conserved intronic site that promotes a complete loss of a canonical splice donor site [46,47]. Finally, mutations in *GM2A* are extremely rare and only 9 mutations have been described on 11 patients [48,49]. Figure 3 show some of the most common mutations on *HEXA*, *HEXB*, and *GM2A* genes.

## 5. Clinical Presentations and Biochemical Correlations of GM2 Gangliosidoses

Although TSD, SD, and AB variant are a consequence of mutations in different genes, the neurological compromise is similar among these three disorders [1]. Classical neurological findings are associated with the clinical onset as follows: (1) *acute*: seizures, hypotonia, regression in developmental milestones [50], (2) *subacute*: motor regression, psychotic episodes, intellectual disability [51], and (3) *chronic*: dysphagia, cerebellar ataxia, muscle weakness, and manic depression [6]. In Table 1 we summarized the neurological findings of GM2 gangliosidoses. In addition, it is important to highlight that SD patients may present systemic manifestations such as cardiomegaly, hepatosplenomegaly, macroglossia, and skeletal abnormalities [7,8]. These systemic manifestations are a consequence of the impaired HexB activity, which is also involved in the degradation of glycosaminoglycans such as keratan sulfate and chondroitin sulfate [52,53].

### Diagnosis of GM2 Gangliosidose

The diagnostic of GM2 gangliosidoses patients begins with recognition of the clinical characteristics of these disorders (Table 1) [7,8,9,58]. Diagnosis is also favored by neuroimaging characterized by hyperdensity of basal ganglia, which can be accompanied by other changes in white matter and sometimes prominent, but non-specific, cerebellar atrophy [9].

The specific diagnosis requires the determination of HexA and HexB activities by using artificial substrates. For instance, the use of 4-methlyumbelliferyl-N-acetylglucosaminide (MUG) allows measurement of the activity of both HexA and HexB, while a sulfated substrate (MUGS) is used to specifically measure the HexA activity [9]. Despite Hex activity can be assessed on biological fluids, cells, tissues, and dried blood spots (DBS) [10], the diagnosis gold standard is the measurement of the enzyme activity in leukocytes, fibroblasts, or chorionic villi [10]. Nevertheless, the β-hexosaminidase activity assay does not allow the identification of asymptomatic carriers or the diagnosis of patients with deficiency on the GM2 activator protein, for who molecular diagnosis is require [9]. Molecular diagnosis by sequencing the *HEXA*, *HEXB,* and *GM2A* genes allows confirmation of the diagnosis of all GM2 gangliosidoses subtypes.

The heterogeneity of onset correlates inversely with the residual catabolic activity of Hex [1,59]. Although patients with the acute presentation have absent or very low (<5%) enzyme activity, patients with subacute or chronic onset may have enzyme activities between 5 and 10% [4,53,60]. In this sense, it has been proposed that a 10% of wild-type enzyme activity may avoid the disease, based on early studies describing GM2 ganglioside degradation with activities between 10 and 15% [61], and pseudo-deficiencies reported in healthy individuals [1,4]. Nevertheless, some pathogenic mutations in both TSD and SD may lead to enzyme activities around 15% of wild-type levels [54,55]. In this sense, it has been proposed that a therapeutic benefit of any of the treatment alternatives for GM2 gangliosidoses may reach enzyme activities higher than 15% of the wild-type levels. Recent findings in this interesting topic will be discussed in detail later.

## 6. Physiopathology of GM2 Gangliosidoses

**Figure 4:**
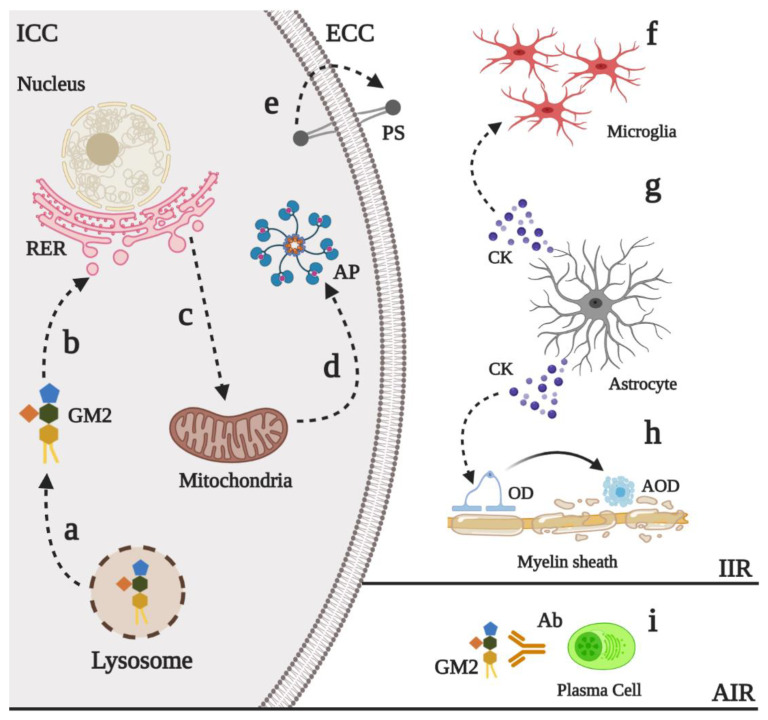
Physiopathological events in GM2 gangliosidoses. Innate (IIR) and adaptative (AIR) immune response have been described in GM2 gangliosidoses. Upon the impaired of lysosomal degradation of the GM2 ganglioside, this can be released into the cytoplasm (**a**) where its sialic acid can interact with the rough endoplasmic reticulum (RER) (**b**). Sustained stress into RER induces the activation of proapoptotic proteins such as CHOP (**c**) that promotes mitochondrial-mediated apoptosis (**d**) In early apoptosis states, the phosphatidylserine (PS) is externalized (**e**), which promotes the recruitment of several proinflammatory cells such as microglia (**f**). Astrogliosis has also been reported in the GM2 gangliosidoses (**g**). The release of several astrocyte-derived cytokines (CK), such as CCL2 and CXCL10, increases the recruitment of microglia and the apoptosis in myelinating oligodendrocytes (AOD), which induces neuron demyelination (**h**). Finally, auto antibodies (Ab) against GM2 ganglioside seem to contribute to the physiopathology of the GM2 gangliosidoses (**i**), although the precise mechanism of its release has not described. ECC: Extracellular compartment, ICC: Intracellular compartment, AP: Apoptosome, OD: Oligodendrocyte.

As a consequence of mutations on Hex subunits or the GM2 activator protein, GM2 gangliosides are accumulated into lysosomes [1,4]. Although it is not completely understood yet, early evidence using animal models of SD showed the presence of autoantibodies against GM2 gangliosides in serum and CNS [62]. This finding may suggest that GM2 ganglioside accumulation could promote the disruption of the lysosome and the release of the ganglioside, as has been described for some mucopolysaccharidoses (MPS) [63]. Since the lysosome has a pivotal function in the degradation of several macromolecules and organelles [64,65,66], its impairment will affect the cellular homeostasis with a negative impact on the global tissue physiology. Here we described the current findings of the physiopathology of these disorders, which are summarized in Figure 4.

### 6.1. Neurodevelopment Process

The use of a cerebral SD organoid model, generated from patient-derived iPS cells, has been an interesting approximation to the discovery of novel implications of GM2 accumulation [67]. In this context, recent findings have described that impaired Hex activity promotes an increase in the size of the cerebral organoids, which was corrected after transduction of *HEXA* and *HEXB* cDNAs by using adeno-associated virus (AAV) vectors [68]. In agreement with these results, and using *HEXB* deficient zebrafish embryos, Kuil et al., 2019 found an increase in the lysosomal speckles in radial glia [69], which are progenitor cells able to difference in neurons, astrocytes, and oligodendrocytes [70]. At 5 days postfertilization, the authors also found reduction in the spherical form of lysosome within microglia, in addition to an abnormal locomotor activity [69]. Nevertheless, an increase in the apoptosis rate was not reported in these studies [68,69]. Together, these results are evidence of the cellular and functional consequences of GM2 ganglioside accumulation in the maturing of the nervous system. These findings also support the fact that the impairment of these cellular processes could have an impact on the acute symptoms of SD, as has been previously identified [71].

### 6.2. Neural Death and Neuroinflammation

Neural death has been proposed as an important mechanism in the physiopathology of the GM2 gangliosidoses [4]. Early studies of brain and spinal cord from autopsy samples of TSD and SD patients revealed an increase in the in situ DNA end-labeling, suggesting that apoptosis could contribute to the neurodegenerative process in these patients [72]. Similar findings were later described in SD animal models [73] and TSD mouse model (*HEXA^-/-^/NEU^-/-^*) [12], showing a marked reduction in neuronal density [74]. Although the precise pathway that explains the increase of neural apoptosis has not been completely resolved, recently findings have shown that GM2 ganglioside can induce endoplasmic reticulum stress [5,75]. It has been also proposed that GM2 ganglioside, but not asialo-GM2 ganglioside, promotes neurite atrophy and cell dead trough PKR-like endoplasmic reticulum kinase (PERK)-mediated apoptosis with downstream CHOP activation [5], which is an inductor of mitochondrial apoptosis [76]. These results suggest that the sialic acid on the GM2 ganglioside may have proapoptotic properties. Nevertheless, the use of a PERK inhibitor did not completely abolish the apoptosis, suggesting that further mechanisms in the neuronal death in GM2 gangliosidoses could be involved [5].

Given the typical mechanism of early apoptosis such as phosphatidylserine externalization on the surface of the neuron [77,78], it has been suggested that the characteristic microgliosis and infiltration observed in patients and animal models may be the response to the neuronal death [4,73,79]. Early studies conducted by Jeyakumar et al., 2003 using a SD (*HEXB^-/-^*) mouse model, showed that as the disease progress and symptoms such as head tremor, motor disfunction, and hind limb paralysis appear, there is an increase in the immunoreactivity for the Mayor Histocompatibility Complex type II in brain stem and thalamus, as well as an increase in the proinflammatory cytokines TNFα and IL1β [80]. These findings suggest that innate immune activation of the CNS could be produced, at least in part, in response to the neuronal death. Although authors reported similar findings on the TSD (*HEXA^−/−^*) mouse model, cytokines levels were not increased. This finding can be explained by the fact that neuraminidase/sialidase catabolize the conversion of GM2 ganglioside to the glycolipid GA2, which is further processed by HexB, thus bypassing the HexA defect [11,12].

Despite microglial activation and proliferation have been extensively reported in TSD and SD models [79,81], astrogliosis can also play an important role in GM2 gangliosidoses even in asymptomatic states of the disease [82,83]. In this regard, Ogawa et al., 2017 showed in SD mice that astrocytes are activated by an FcRγ-dependent mechanism [83]. Upon activation, astrocyte secretes the chemokines CCL2 and CXCL10, which correlates with a robust microglial activation and the invasion of peripheral immune cells. This astrocytes activation could lead to degeneration and death of myelinating oligodendrocytes, promoting active demyelination [84,85], which is frequently observed in infantile forms of GM2 gangliosidoses [4,86,87]. Together, these findings highlight the pivotal interplay between microglia and astrocytes in the inflammatory response observed in GM2 gangliosidoses, which could be the functional consequence of neuronal injury due to GM2 ganglioside accumulation and that may contribute to the neurodegenerative process.

## 7. Current Proposals for the Treatment of GM2 Gangliosidoses

**Figure 5:**
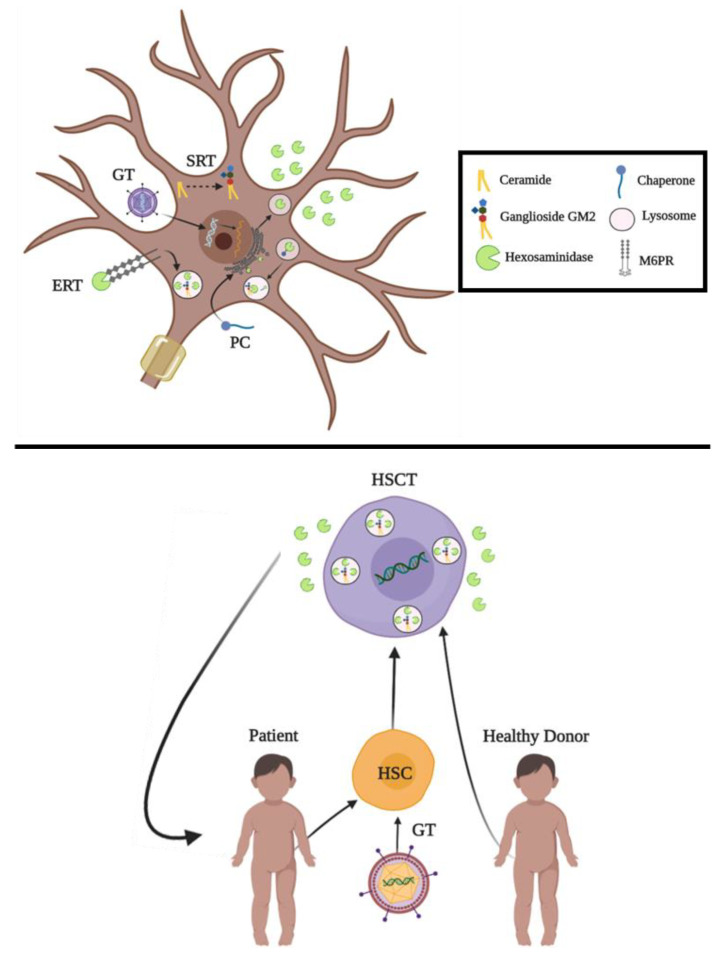
Therapeutic alternatives for GM2 gangliosidoses. The figure shows current proposals for in vivo (**upper**) and ex vivo (**lower**) approaches. Extracellular Hex represents exocytosis of the enzyme upon its translation, which supports the cross-correction hypothesis. GT: Gene Therapy. SRT: Substrate Reduction Therapy. PC: Pharmacological Chaperones. ERT: Enzyme Replacement Therapy. HSCT: Hematopoietic Stem Cell Transplantation. HSC: Hematopoietic Stem Cell.

Several approaches have been tested for the development of specific treatments for GM2 gangliosidoses, which are summarize in Figure 5 [88,89,90]. These strategies range from traditional enzyme replacement therapy alternatives to novel biotechnological tools such as CRISPR/Cas9 and prime editing, with promising results both in vitro and in vivo. Although some of these developments have been translated to clinical trials [91,92,93,94] (Table 2), there is not an approved treatment for TSD, SD, or AB variant yet. In this section, we review the current proposals and advances in therapeutics for GM2 gangliosidoses.

### 7.1. Enzyme Replacement Therapy

Enzyme replacement therapy (ERT) is a therapeutic alternative conceived in 1964 by Christian de Duve in which the lysosomal enzymes can be uptake through endocytosis and delivered to the lysosomes [14]. ERT is possible due to the cell uptake of mannosylated enzymes via specialized mannose receptors [95]. Currently, ERT has been approved for Gaucher, Fabry, and Pompe diseases, late infantile neuronal ceroid lipofuscinosis type II, acid lipase deficiency, alpha-mannosidosis, and MPS type I, II, IVA, VI, and VII [96].

In the case of GM2 gangliosidoses, early studies carried out by Johnson et al., 1973 using intravenous administration of HexA in a patient with SD showed that the enzyme was presented in liver but not in the cerebrospinal fluid or brain parenchyma, suggesting that enzyme was unable to cross the BBB [97]. To overcome these limitations, promising strategies to enable that exogenous lysosomal enzymes can cross the BBB include the development of fusion proteins, also known as molecular Trojan horses [14]. These fused proteins are recombinant chimeric enzymes fused to a monoclonal antibody (MAb) that recognize either the human insulin receptor (HIR) or the transferrin receptor (TfR), and which allow the passage of the BBB through a receptor-mediated endocytosis [88]. In this field, several studies using non-human primates have shown that intravenous administration of HIRMAb fused to α-iduronidase [89], iduronate-2-sulphatase [90], sulfamidase [91], α-N-acetylglucosaminidase [92] can cross the BBB without side effects. Although these approaches have not been evaluated in animal models of GM2 gangliosidoses, recently Boado et al., 2019 showed that a HexA fused to HIRMAb has a similar activity to the non-fused enzyme (2.464 ± 109 mU/mg vs. 2.557 ± 187 mU/mg, respectively), suggesting that use of molecular Trojan horses could be an alternative to treat GM2 gangliosidoses [88].

The use of several routes such as direct injection into the cerebrospinal fluid and intrathecal or intracerebroventricular (ICV) injections have shown potential therapeutic effects for some LSDs with CNS compromise [14,93,94]. In this sense, it has been reported the ICV administration to SD mice of recombinant HexA produced in Chinese hamster ovary (CHO) cells or the yeast *Ogataea minuta*, with or without modifications on the N-glycans [4,98,99]. The results showed significant increase on the enzyme activity levels in several brain regions and in the life spam, as well as a reduction on the levels of GM2 and GA2 gangliosides and the MIP-1α chemokine [4,98,99]. Similarly, Matsuoka et al., 2011 evaluated the ICV administration of a chimeric HexB enzyme, which contain a β-subunits with six point mutations and a partial sequence from the α-subunit that allows the binding of charged substrates and GM2AP [100]. This approach, led to a significant reduction of GM2 ganglioside in the brain and cerebellum and a 2-fold increase in Hex activity. Noteworthy, a marked reduction of GM2 ganglioside was also observed in liver; which could have a significant impact on the disease, since hepatosplenomegaly is a common clinical finding of SD patients [87]. Together, these results suggest that ICV ERT is a potential strategy for the treatment of GM2 gangliosidoses and that N-glycans modifications may have a positive effect on enzyme uptake, biodistribution, and substrate reduction throughout the brain.

Although most of the enzymes used for ERT are produced in CHO cells, recombinant Hex produced in the yeast *O. minuta* and *Pichia pastoris* have also been evaluated [99,101,102]. In this sense, recombinant HexA and HexB produced in the methylotrophic yeast *Pichia pastoris* GS115 have shown high pH and serum stability [101]. In addition, these enzymes are internalized by an M6PR-dependent mechanism without the need for further modifications of the N-glycans [103], unlike that reported for the recombinant HexA produced in *O. minuta* [99]. In vitro evaluation of recombinant HexA produced in *P. pastoris* showed normalized lipid accumulation in neural stem cells, showing the potential of this enzyme in the development of an ERT for GM2 gangliosidoses [104]. Recent efforts to allow the delivery of these recombinant Hex to CNS involved their conjugation with nanoparticles [105].

### 7.2. Hematopoietic Stem Cell Transplantation

Since Hex can be exported to the extracellular space [37,45] and cross-correct neighboring cells through a M6PR-mediated mechanism [4,106], the administration of hematopoietic stem cells (HSC), could provide sufficient amounts of the deficient enzyme in a natural o engineered-dependent manner [106,107]. Allogenic HSC transplantation (HSCT) can be performed from bone marrow (BM), umbilical cordon (UC), or peripheral blood after a myeloablative regimen [108]. This strategy has been successfully performed in other LSDs such as MPS I [109,110], MPS II [111], and Gaucher type 1 and 2 [112].

For GM2 gangliosidoses a limited number of patients have been subject to HSCT. In this regard, Jacobs et al., 2005, used allogeneic BM transplantation to treat a 3 years-old asymptomatic child with subacute TSD. The results showed an increase in leukocytes HexA activity without prevention of the neurodegenerative events of the disease [113]. Later, in a single-center study, five children with infantile TSD were subjected to unrelated UC transplantation. After treatment, the survival was extended in 2 cases with an arrest on the neurodegenerative process, without improvement in motor skills [114].

Recently, Ornaghi et al., 2020 used lentiviral vectors carrying both α- and β-subunits to transduce HSC isolated from healthy donors, as well as neural stem cells and murine HSC, showing an increase of up to 2-fold in the total Hex activity [115]. In addition, physiological functions of these stem cells, such as proliferation, self-renewal, or multipotency, remain unchanged, suggesting that ex vivo gene therapy could be an interesting option for the treatment of GM2 gangliosidoses [115]. Nevertheless, a common challenge in allogeneic HSCT is the graft-versus-host disease (GVHD) [116]. In fact, studies in patients with inborn errors of metabolism have shown up to 10% of acute GVHD [114]. To overcome this limitation, recent developments using hypoimmunogenic human stem cells have opened a new horizon for the HSCT, avoiding the events of GVHD through evasion of both cellular and humoral immune response [117,118]. However, this approach has not been applied to GM2 gangliosidoses yet. Likewise, an attractive alternative for the treatment of GM2 gangliosidoses could be the use of autologous HSCT, in which the mutations are corrected thorough gene therapy in hematopoietic precursors isolated from the patients [107,119].

### 7.3. Pharmacological Chaperones

Pharmacological chaperones (PCs) are small molecules that bind to a target protein stabilizing the native conformation or promoting the correct folding [120,121]. PCs bind with high affinity and selectivity to the target protein into the ER and later dissociated into the lysosome as a consequence of the acidic pH and the presence of the natural substrate, which competes with the PC for the active site [122,123]. Since most of the PC described so far act as competitive inhibitors [124], it has been proposed the use of allosteric sites to identify non-inhibitory PC [122]. In addition, PCs have a mutation-dependent activity, which limits the number of patients that may respond to the treatment [122,123]. PCs must not be confused with chemical chaperones such as dimethyl sulfoxide (DMSO), which can also bind to and stabilize some proteins but with less selectivity and greater toxicity [125].

In a screening of 1040 FDA-approved drugs, Maegawa et al., 2007 found that pyrimethamine (PYR) was the most promising PC for HexA since it induced an up to 3-fold increase in enzyme activity on TSD fibroblasts [126,127]. PYR is a drug used in the treatment of cerebral toxoplasmosis and complicated malaria, which targets folic acid synthesis and can cross the BBB [128]. Nevertheless, clinical trials with PYR showed an increase in enzyme activity but with a limited impact on the CNS manifestations of the disease [4,129,130].

To identify potential PCs for GM2 gangliosidoses, an in silico analysis of iminosugar inhibitors that bind to the active site of HexA was carried out [131]. Throughout molecular docking and dynamics simulations, the pyrrolidine 2,5-dideoxy-2,5-imino-D-mannitol (DMDP) amide was predicted as the strongest competitive inhibitor of HexA. DMDP amide improved the intracellular activity of HexA up to 14.8-fold in TSD fibroblasts patients, reaching up to 43% of wild-type levels [131]. These results suggest that iminosugars could be an interesting therapeutic alternative for GM2 gangliosidoses, as has been previously described for other LSDs [122,124].

Another potential strategy reported to increase the folding of mutant Hex is the use of progranulin (PGRN). This is a glycoprotein secreted by epithelial, neuronal, and immune cells that is involved in a variety of physiological processes and diseases such as early embryogenesis, cell proliferation, immune and neurodegenerative diseases, inflammation processes, wound healing, and tissue repair [132,133]. Jian et al., 2016 showed that the heat shock protein 70 associates with the lysosomal enzyme β-glucocerebrosidase (GCase) in the ER/Golgi apparatus by a PGRN-dependent manner, avoiding the aggregation of the GCase [133]. Since PGRN could be considered to be a co-chaperone, it was evaluated to increase the HexA activity in fibroblasts from TSD patients. The results showed that the G and E domains of the PGRN bind to HexA, increasing the enzyme activity and lysosomal delivery, and leading to a GM2 reduction in TSD fibroblasts [132].

### 7.4. Substrate Reduction Therapy

Substrate reduction therapy (SRT) is a therapeutic strategy based on the partial inhibition of an enzyme involves in the synthesis of the accumulating substrate [134]. One of the molecules that have been evaluated for this purpose is N-butyldeoxynojirimycin (NB-DNJ, also termed Miglustat or Zavesca) [135]. This is an iminosugar that inhibits the glucosylceramide synthase (GCS), which catabolize the first step of glycosphingolipid synthesis such as glucosylceramide (Gaucher disease) [136], sphingomyelin (Niemann-Pick type C) [137], GM1 (GM1 gangliosidoses) [138] and GM2 gangliosides [139]. Miglustat is an oral drug able to cross the BBB, slowing the accumulation of gangliosides in neurons, and delaying the progression of neurological symptoms [134]. Miglustat has been evaluated in murine models of SD [140] and TSD [141] with promissory results regarding ganglioside storage reduction, the decrease of neurological symptoms, and the extension of lifespan. Subsequent studies evaluated the effect of miglustat in five patients with juvenile GM2 gangliosidoses in advanced disease stage over a period of 24 months, without improvement on the neurological impairment [142]. Likewise, Masciullo et al., 2010 showed that a 3-years treatment with miglustat on an adult with chronic SD, did not arrest the neurodegeneration [143]. In this sense, implementation of miglustat in early disease stages should be assessed, to understand the therapy efficacy during early intervention.

Ashe et al., 2011 evaluated the molecule Genz-529468, which has an IC_50_ 250-fold greater than miglustat [144]. Genz-529468 was administrated to SD mice and the results were compared with miglustat-treated mice. The effect of each drug on brain GM2 levels was opposite. In this sense, whereas Genz-529468 increased GM2 levels to about 120% of untreated mice; miglustat decreased the GM2 levels to about 90% of untreated mice. However, levels of other brain glycosphingolipids, especially GL1, were dramatically increased with both inhibitors. The authors also reported similar results for both drugs in terms of the delayed loss of motor function and extended of the lifespan. In addition, mice treated with miglustat and Genz-529468 showed lower microglia activation and astrogliosis, and delay of neuronal apoptosis. These findings suggest that the use of inhibitors of GCS could improve the clinical outcomes due, at least in part, to their an anti-inflammatory properties [4,145].

Finally, Arthur et al., 2013 reported the evaluation of EtDO-PIP2, a GCS inhibitor, on juvenile SD mice [146]. The intraperitoneal administration of EtDO-PIP2 to SD mice reduced the total content of brain and liver gangliosides, suggesting that it could be an interesting alternative for the treatment of ganglioside storage diseases with CNS manifestations.

### 7.5. Gene Therapy

Since GM2 gangliosidoses are monogenic diseases, the delivery of a functional gene should correct the genetic defect and lead to a normal physiological development [147]. In recent years, several strategies based on gene therapy have been developed for LSDs, including GM2 gangliosidoses [15,147,148,149], which will be discussed below.

Usually, gene therapy uses recombinant viral vectors to deliver transgenes into specific tissues, from which the AAV-derived vectors have gained great attention during recent years [4]. Nevertheless, AAV can induce an immune response that can limit the efficacy of the therapy [150]. Therefore, new viral vectors have been engineered to modify the viral capsid and reduce the immune response [151,152].

Several papers have described a variety of studies of gene therapy applied to the brain of small and larger animal models of GM2 gangliosidoses, mostly using AAV vectors [4]. Early gene therapy studies for GM2 gangliosidoses were based on the intracranial co-administration of AAV1 or AAVrh8 vectors carrying the α and β subunits on SD mice [153,154] or cats [82,155,156,157]. These results showed a wide distribution of the enzyme through the brain, a significant increase in the life spam, the reduction of GM2 ganglioside levels, and an improvement on motor functions [82,155,156,157,158,159]. Moreover, treated SD cats showed an increase on the myelin-enriched cerebrosides and a decrease in the microglial activation, suggesting an attenuation of the neuroinflammation [79,160], which is an important physiopathology feature observed in GM2 patients [73,80,84]. As an alternative to the multi-vector injections, a self-complementary AAV9.47 vector was evaluated, which encoded for a hybrid µ subunit (HexM) that combine the α-subunit active site, the stable β-subunit interface, and unique areas of each subunit necessary for the interaction with the GM2AP [40,154]. The results showed that HexM was able reduce the GM2 ganglioside storage in thalamus, hypothalamus, and hippocampus; and led to an improvement on animal behavior [154].

During recent years, gene therapy studies for GM2 gangliosidoses have been included the evaluation on larger animal models and the design of bicistronic vectors. For instance, Golebiowski et al. 2017 evaluated the effect of the intracranial administration of AAVrh8 vectors carrying the cynomolgus macaques Hex subunits (α and β) on non-human primates [161]. Although supraphysiological Hex activity levels were achieved in CNS; most of the treated animals showed altered neurological function represented by ataxia, general weakness, and lethargy; as well as histopathological findings suggestive of neurotoxicity [161]. These findings suggest a response not only against vector but also to the supraphysiological levels of Hex, which contrasts to the results observed in other species such as mice or cats. These aspects could be better resolved with similar pre-clinical testing but using validated non-human primates’ models for GM2 gangliosidoses to understand the real therapeutic effect on species close to human.

Recently, Gray-Edwards et al., 2018 used AAVrh8 vectors carrying the α and/or β subunits to evaluate the therapeutic effect of an intracranial injection in a TSD sheep model [81]. The treated TSD sheep achieved wild-type or even supraphysiological levels of HexA activity and a decrease in disease progress. In addition, treated TSD sheep showed a normalization of accumulated gangliosides in most areas of the brain, except cerebellum and spinal cord where the storage did not change. Noteworthy, neuroinflammation was also attenuated after the treatment [81].

Novel approaches using bicistronic vectors have been developed [162]. As a proof-of-concept the bicistronic ssAAV9-HexBP2A-HexA vector was evaluated, which has a short P2A linker and the cDNA of *HEXA* and *HEXB* genes under the control of the chicken β-actin promoter [163]. A single administration of the vector into neonatal SD mice allowed a 56% extension of the lifespan compared to untreated animals. In addition, this vector allowed a significant increase in the enzyme activity and the reduction of GM2 gangliosides levels both in brain and serum [163]. Using similar bicistronic vector but with a bidirectional promoter to drive the transgene expression in opposite directions of the *HEXA* and *HEXB* subunits, recently Lahey et al., 2020 found an increase the survival from 138 for untreated SD mice to up to more than 600 days after of the IV administration of the vectors [41]. Improvement in the motor, strength, and coordination tests for treated animal to similar values observed in wild-type mice was also observed. These promising findings were accompanied by increased levels of HexA and HexB enzyme activity in liver and CNS; as well as reduction in GM2 ganglioside storage in cerebrum, cerebellum, brain stem, and spinal cord of SD mice; which was correlated with a significant reduction of microglial activation in brain [41]. Finally, similar observations were reported for bicistronic lentiviral vectors carrying murine or human cDNAs encoding for the α- and β-subunits linked by a P2A linker. These lentiviral vectors led to a Hex activity up to 5-fold higher than wild-type levels in murine neurons and human stem progenitor cells, as well as a 30–60% reduction of GM2 storage. Similarly, in SD fibroblasts the total Hex and HexA activity increased in an 8:1 ratio, respectively [115].

### 7.6. CRISPR/Cas9-Based Gene Therapy

**Figure 6:**
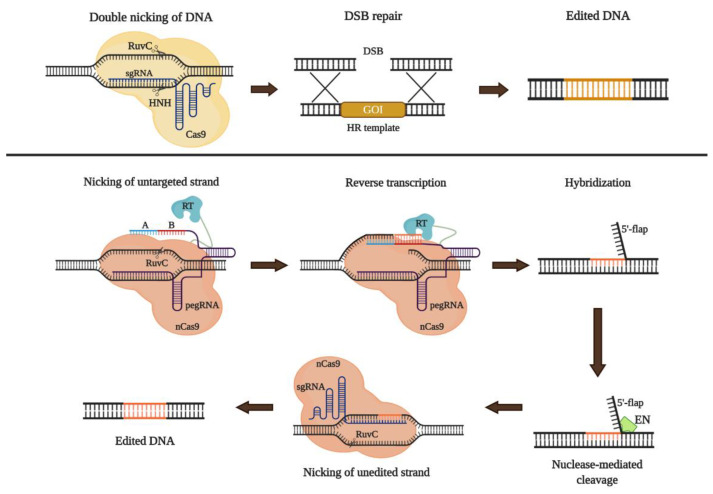
Approaches for genome editing using CRISPR/Cas9. The upper panel shows the classical strategy of knock-in using a ribonucleoprotein complex (sgRNA-guide Cas9) to guide to Cas9 to the target DNA and cut the double-strand (DSB). After the DSB, repair machinery is activated. In the presence of a donor sequence (HR template), homologous recombination is favored. To promote the recombination event of a gene of interest (GOI), the HR template must be flanked by homologous recombination arms which are complementary to the 5′ and 3′-ends of the sequence into the gene that will be subject of edition. Typically, between 100-150bp and 400-800bp are suitable for small (<50 bp) and large (>100) insertions, respectively [165,166]. In the lower panel, *Prime Editing* (PE) is represented. PE uses a nickase Cas9 (nCas9-H840A) fused to reverse transcriptase (RT) and a guide RNA (pegRNA) which is engineered with a sequence in the 5′-end.20 nucleotides guide to nCas9 to the target DNA and a sequence in the 3-end with a primer-binding site (A) as well as an RT template (B) that could be between 7 to 12 nucleotides [167]. Upon reverse transcription, newly synthesized strand hybridizes to the unedited strand (US) forming a mismatch and a 5′-flap strand which is removed by exonucleases (EN) such as EXO1 [168]. The mismatch is resolved with the introduction of a new nCas9 coupled to a simple sgRNA which guide to nCas9 to the edited strand (ES), about 50 bp from the pegRNA-mediated nick, to cut the US and use the sequence of the ES as a template for repair de simple cut [167]. In both cases upper and lower panels, newly edited DNA is successfully obtained with different efficiencies.

Three major genome editing tools have been developed: zinc finger nucleases (ZFN), transcription activator-like effector nucleases (TALEN), and more recently CRISPR/Cas9 [158,164]. CRISPR/Cas9 uses an RNA-guide nuclease (sgRNA-Cas9) to induce double-strand breaks (DSB) into a specific locus of the genome [155,159]. DSB can be repaired either through non-homologous end joining (NHEJ) or homologous direct repair (HDR) pathways [15]. On the first case, upon the DSB and on absence of a DNA template, the cellular repair machinery recruits several effectors such as Ku70/80, DNA-PKcs, Artemis, and DNA ligase IV that promote the binding of non-homologous ends in an error-prone mechanism. Through this mechanism is possible to induce deletions or insertions leading to insertional inactivation [156]. This approach is used to knock-out target genes, and has been used to generate in vitro and in vivo models of GM2 gangliosidoses [40,68,69]. DSB can also be repaired through the HDR pathway mechanism by using an exogenous DNA fragment as template (donor DNA), to mediates the insertion of the therapeutic DNA fragment on a specific locus (Figure 6) [156,157,158].

Although the punctual correction of each mutation could be interesting, in pathologies with hundreds of mutations, such as GM2 gangliosidoses, this approach is not suitable. As a consequence, the knock-in strategy using safe harbors to introduce the cDNA into the genome draws more attention [169,170]. Ou et al., 2020 recently used the albumin locus for the intravenous administration of two AAV8 vectors: one carrying a promoterless HexM cDNA, and other one carrying the Cas9 gene and the gRNA [171]. This approach allowed the constitutive expression of the HexM under the control of the albumin promoter. Four-months post-treatment, the enzyme activity increased in plasma, heart, liver, spleen, and brain, compared to untreated SD mice. This increase in the HexM activity correlated with the decrease of the GM2 ganglioside in liver, heart, and spleen. On the other hand, neither a decrease of GM2 ganglioside in the brain nor positive changes on behavioral tests (fear conditioning and pole test) were observed as a result of the treatment [171]. Despite the above, a significant improvement motor function was observed in treated SD mice compared to untreated animals suggesting a slight therapeutic effect on the CNS. Overall, these results show that although this strategy must be optimized, the insertion of HexM gene into hepatocytes is an alternative to reach supraphysiological levels of the enzyme, particularly into the brain, where typical strategies such as ERT fails due to the inability to cross the BBB [13]. Since new epitopes can be generate from HexM, immune tolerance regimens must be also considered [41].

Despite HDR is the main approach used in CRISPR/Cas9-mediated genome editing, novel strategy without DBS or donor DNA, termed prime editing, was recently described [172,173]. This tool uses a nickase Cas9 (H840A) fused to reverse transcriptase (RT) and a short engineered RNA sequence (prime editing guide RNA-pegRNA) [167]. The pegRNA is designed to function as a guide of Cas9 to the target DNA and serve as the template for a RT-mediated retrotranscription, avoiding the need for a DNA donor [172,174]. An additional sgRNA-Cas9 is necessary to nicking the unedited strand and promotes the final correction using the edited strand as a template, in a process that occurs only after the edition and avoiding the generation of DSB [167,174] (Figure 6). Using this strategy, Anzalone et al., 2019 recreated in HEK293T cells a 4-bp insertion in *HEXA* gene (1278 + TATC), which is associated with TSD, with high efficiency (31%) and low indels (0.8%). The cells were then correct using the prime editing strategy, thorough the evaluation of 43 pegRNA and three sgRNA. Nineteen pegRNAs showed edition efficiencies higher than 20% and lower indels 0.32% [167], suggesting that this novel strategy could be a new therapeutic approach for the treatment of GM2 gangliosidoses without the need for DSB or donor template.

## 8. Conclusions and Perspectives

During recent years significant advances have been done to understand the physiopathology and natural history of GM2 gangliosidoses. For instance, natural history programs have allowed to identify the shared and specific manifestations of each disease and phenotype (infantile/acute, juvenile/subacute, and adult/chronic), which represent valuable information to improve the diagnosis and patients follow up. However, important efforts still need to be done in order to include a wider number of patients with different genetic backgrounds, since most of the studies have been carried out in specific populations (e.g., clinicaltrials.gov NCT01869270, NCT02851862, NCT00668187, NCT03333200, and NCT00029965).

On the other hand, although gangliosides remain as the main compounds responsible for neuron homeostasis alteration, neuroinflammation and demyelination have also shown to play an important role in disease progression. In addition, the presence of anti-ganglioside autoantibodies, progressive accumulation of α-synuclein, and impaired autophagy, are elements that have been also associated with the disease process [4]. In this sense, as observed in other LSDs [175], it is possible that a single therapeutic strategy would not be enough to treat GM2 gangliosidoses patients and that the co-administration of different therapies may be required. Noteworthy, different therapeutic strategies, including ERT, gene therapy, PCs, HSCT, and SRT, have shown promising results and some of them have reached clinical phases (Table 2). In addition, the National Tay–Sachs, and Allied Diseases (https://ntsad.org/) announced the beginning of gene therapy clinical trials for GM2 gangliosidoses. Although the use of CRISPR/Cas9 is still on its initial stages, the first pre-clinical studies have shown the potential of this tool in the design of novel gene therapy strategies.

Finally, epigenetics should be also considered in GM2 gangliosidoses. Although the role of epigenetic mechanisms in lysosomal diseases has not been well stablished, it has been proposed that these mechanisms may contribute to the clinical heterogeneity observed in these disorders [176]. However, to the best of our knowledge, no studies on this field has been performed for GM2 gangliosidoses. The understanding of the epigenetic alterations observed in GM2 gangliosidoses patients may represent an opportunity to the develop of novel treatment alternatives [177].

## Figures and Tables

**Table 1:** Main neurological features in GM2 gangliosidoses.

Disease	Affected Gene	Affected Protein	Accumulated Substrate *	Common Findings			Ref
				Onset	Symptoms	Neuroimaging	
**TSD**	*HEXA*	HexA	GM2 Ganglioside	**Infantile Acute**	Seizures, axial hypotonia, cherry-red spot, regression in developmental milestones, exaggerated startle response	Bilateral thalamic involvement, brain atrophy, hypomyelination	[50,54,55]
**SD**	*HEXB*	HexA, HexB	GM2 Ganglioside, Globoside	**Juvenile Subacute**	Ataxia, myoclonus, motor regression, psychotic episodes, intellectual disability, progressive clumsiness	Cerebellar atrophy	[51,56]
**AB Variant**	*GM2A*	GM2AP	GM2 Ganglioside	**Adult Chronic**	Dysphagia, muscle atrophy, cerebellar ataxia, dysarthric speech, manic depression, muscle weakness, psychotic episodes	Severe cerebellar atrophy, hypodensity of the thalamus	[4,6,57]

TSD: Tay–Sachs disease SD: Sandhoff disease. * Note: β-Hexosaminidases can hydrolyze molecules with N-acetyl-hexosamines residues such as glycosaminoglycans (GAGs). Accumulation of partially degraded GAGs has also been reported. For more detail please see [52,58].

**Table 2:** Clinical trials for GM2 gangliosidoses reported at Clinicaltrials.gov by June 2020.

Therapy	NCT Number	Intervention	Status	Phase	Country
**Pharmacological Chaperone**	NCT00679744	Pyrimethamine	Withdrawn	Phase 1	USA
	NCT01102686	Pyrimethamine	Completed	Phase 1/2	Canada
**Substrate Reduction Therapy**	NCT00418847	Miglustat	Completed	Phase 2	Canada
	NCT03822013	Miglustat	Recruiting	Phase 3	Iran
	NCT00672022	Miglustat	Completed	Phase 3	USA
	NCT04221451	Venglustat	Recruiting	Phase 3	USA
	NCT02030015	Miglustat and ketogenic diet	Recruiting	Phase 4	USA
**HSCT**	NCT01372228	Enriched hematopoietic stem cell infusion	Active, not recruiting	Phase 1/2	USA
	NCT00176904	Chemotherapy and hematopoietic cell transplantation	Completed	Phase 1/2	USA
	NCT01626092	Chemotherapy, total body irradiation with marrow boosting, and hematopoietic stem cell transplantation	Completed	Phase 1/2	USA
	NCT00383448	Chemotherapy, total body irradiation, and hematopoietic stem cell transplantation	Completed	Phase 2	USA
**Umbilical Cord Blood Transplantation (UBC)**	NCT02254863	UBC-derived oligodendrocyte-like cells	Recruiting	Phase 1	USA
	NCT01003912	Fetal UCB transplantation	Withdrawn	Phase 1	USA
	NCT00654433	UBC cells expressing high levels of the intracellular enzyme aldehyde dehydrogenase	Terminated	Phase 3	USA
**Cerebellar Ataxia Treatment**	NCT03759665	N-Acetyl-L-Leucine	Recruiting	Phase 2	USA

UCB: umbilical cordon blood.

## Abbreviations

AAV	Adeno-associated virus
Ab	Antibodies
AIR	Adaptative immune response
AOD	Apoptotic oligodendrocytes
AP	Apoptosis
BBB	Blood–brain barrier
Cas9	CRISPR-associated protein 9
CK	Cytokines
CNS	Central Nervous System
CRISPR	Clustered Regularly Interspaced Short Palindromic Repeats
DMDP	2,5-dideoxy-2,5-imino-D-mannitol
DMSO	Dimethyl sulfoxide
DOAJ	Directory of open access journals
DSB	Double-strand break
ECC	Extracellular compartment
EE	Early endosome
ERT	Enzyme Replacement Therapy
FDA	Food and Drug Administration
GAG	Glycosaminoglycans
GCS	Glucosylceramide synthase
GluCer	Glucosylceramide
GM2-AP	GM2-activator protein
GT	Gene therapy
GVHD	Graft-versus-host disease
HDR	Homologous direct repair
Hex	Hexosaminidase
HLA	human leukocyte antigen
HSCT	Hematopoietic Stem Cell Transplantation
ICC	Intracellular compartment
IIR	Innate immune response
iPS	Induced pluripotent stem
LacCer	Lactoceramide
LD	Linear dichroism
LSD	Lysosomal Storage Disorders
M6PR	Mannose-6 phosphate receptor
MDPI	Multidisciplinary Digital Publishing Institute
MPS	Mucopolysaccharidosis
MUG	4-methlyumbelliferyl-N-acetylglucosaminide
MUGS	4-methylumbelliferyl-beta-D-N-acetyl-glucosamine-6-sulfate
NHEJ	Non-homologous end joining
OD	Oligodendrocytes
PC	Pharmacological Chaperones
PERK	PKR-like endoplasmic reticulum kinase
PGRN	Progranulin
PS	Phosphatidylserine
PYR	Pyrimethamine
RER	Rough endoplasmic reticulum
SD	Sandhoff disease
SRT	Substrate Reduction Therapy
TGN	Trans-Golgi network
TSD	Tay–Sachs disease
UCB	Umbilical cordon blood

## References

[1] Sandhoff K., Harzer K. (2013). Gangliosides and Gangliosidoses: Principles of Molecular and Metabolic Pathogenesis. J. Neurosci..

[2] Schnaar R.L. (2019). The Biology of Gangliosides. Adv. Carbohydr. Chem. Biochem..

[3] Ledeen R., Wu G. (2018). Gangliosides of the Nervous System. Methods Mol. Biol..

[4] Cachon-Gonzalez M.B., Zaccariotto E., Cox T.M. (2018). Genetics and Therapies for GM2 Gangliosidosis. Curr. Gene Ther..

[5] Virgolini M.J., Feliziani C., Cambiasso M.J., Lopez P.H., Bollo M. (2019). Neurite Atrophy and Apoptosis Mediated by PERK Signaling after Accumulation of GM2-Ganglioside. Biochim. Biophys. Acta Mol. Cell Res..

[6] Masingue M., Dufour L., Lenglet T., Saleille L., Goizet C., Ayrignac X., Ory-Magne F., Barth M., Lamari F., Mandia D. (2020). Natural History of Adult Patients with GM2 Gangliosidosis. Ann. Neurol..

[7] Jain A., Kohli A., Sachan D. (2010). Infantile Sandhoff’s Disease with Peripheral Neuropathy. Pediatr. Neurol..

[8] Venugopalan P., Joshi S.N. (2002). Cardiac Involvement in Infantile Sandhoff Disease. J. Paediatr. Child Health.

[9] Hall P., Minnich S., Teigen C., Raymond K. (2014). Diagnosing Lysosomal Storage Disorders: The GM2 Gangliosidoses. Curr. Protoc. Hum. Genet..

[10] Zhang J., Chen H., Kornreich R., Yu C. (2019). Prenatal Diagnosis of Tay-Sachs Disease. Methods Mol. Biol..

[11] Lawson C.A., Martin D.R. (2016). Animal Models of GM2 Gangliosidosis: Utility and Limitations. Appl. Clin. Genet..

[12] Seyrantepe V., Demir S.A., Timur Z.K., Von Gerichten J., Marsching C., Erdemli E., Oztas E., Takahashi K., Yamaguchi K., Ates N. (2018). Murine Sialidase Neu3 Facilitates GM2 Degradation and Bypass in Mouse Model of Tay-Sachs Disease. Exp. Neurol..

[13] Concolino D., Deodato F., Parini R. (2018). Enzyme Replacement Therapy: Efficacy and Limitations. Ital. J. Pediatr..

[14] Giugliani R., Vairo F., Kubaski F., Poswar F., Riegel M., Baldo G., Saute J.A. (2018). Neurological Manifestations of Lysosomal Disorders and Emerging Therapies Targeting the CNS. Lancet Child Adolesc. Health.

[15] Leal A.F., Espejo-Mojica A.J., Sánchez O.F., Ramírez C.M., Reyes L.H., Cruz J.C., Alméciga-Díaz C.J. (2020). Lysosomal Storage Diseases: Current Therapies and Future Alternatives. J. Mol. Med..

[16] Yu R.K., Tsai Y.T., Ariga T., Yanagisawa M. (2011). Structures, Biosynthesis, and Functions of Gangliosides—An Overview. J. Oleo Sci..

[17] Prokazova N.V., Samovilova N.N., Gracheva E.V., Golovanova N.K. (2009). Ganglioside GM3 and its Biological Functions. Biochemistry.

[18] Aureli M., Mauri L., Ciampa M.G., Prinetti A., Toffano G., Secchieri C., Sonnino S. (2016). GM1 Ganglioside: Past Studies and Future Potential. Mol. Neurobiol..

[19] Riboni L., Malesci A., Gaini S.M., Sonnino S., Ghidoni R., Tettamanti G. (1984). Ganglioside Pattern of Normal Human Brain, from Samples Obtained at Surgery. A Study Especially Referred to Alkali Labile Species. J. Biochem..

[20] Zeller C.B., Marchase R.B. (1992). Gangliosides as Modulators of Cell Function. Am. J. Physiol..

[21] Sonnino S., Chiricozzi E., Grassi S., Mauri L., Prioni S., Prinetti A. (2018). Gangliosides in Membrane Organization. Prog. Mol. Biol. Transl. Sci..

[22] Lopez P.H.H., Báez B.B. (2018). Gangliosides in Axon Stability and Regeneration. Prog. Mol. Biol. Transl. Sci..

[23] Todeschini A., Hakomori S.I. (2008). Functional Role of Glycosphingolipids and Gangliosides in Control of Cell Adhesion, Motility, and Growth, through Glycosynaptic Microdomains. Biochim. Biophys. Acta Gen. Subj..

[24] Groux-Degroote S., Rodríguez-Walker M., Dewald J.H., Daniotti J.L., Delannoy P. (2018). Gangliosides in Cancer Cell Signaling. Prog. Mol. Biol. Transl. Sci..

[25] Rubovitch V., Zilberstein Y., Chapman J., Schreiber S., Pick C.G. (2017). Restoring GM1 Ganglioside Expression Ameliorates Axonal Outgrowth Inhibition and Cognitive Impairments Induced by Blast Traumatic Brain Injury. Sci. Rep..

[26] Kolter T. (2012). Ganglioside Biochemistry. ISRN Biochem..

[27] Lutz M.S., Jaskiewicz E., Darling D.S., Furukawa K., Young W.W. (1994). Cloned Beta 1,4 N-Acetylgalactosaminyltransferase Synthesizes GA2 as Well as Gangliosides GM2 and GD2. GM3 Synthesis has Priority over GA2 Synthesis for Utilization of Lactosylceramide Substrate In Vivo. J. Biol. Chem..

[28] Mahuran D.J. (1999). Biochemical Consequences of Mutations Causing the GM2 Gangliosidoses. Biochim. Biophys. Acta.

[29] Hasilik A., Neufeld E.F. (1980). Biosynthesis of Lysosomal Enzymes in Fibroblasts. Phosphorylation of Mannose Residues. J. Biol. Chem..

[30] Proia R.L., Soravia E. (1987). Organization of the Gene Encoding the Human Beta-Hexosaminidase Alpha-Chain. J. Biol. Chem..

[31] Little L.E., Lau M.M., Quon D.V., Fowler A.V., Neufeld E.F. (1988). Proteolytic Processing of the Alpha-Chain of the Lysosomal Enzyme, Beta-Hexosaminidase, in Normal Human Fibroblasts. J. Biol. Chem..

[32] Quon D.V., Proia R.L., Fowler A.V., Bleibaum J., Neufeld E.F. (1989). Proteolytic Processing of the Beta-Subunit of the Lysosomal Enzyme, Beta-Hexosaminidase, in Normal Human Fibroblasts. J. Biol. Chem..

[33] Lemieux M.J., Mark B.L., Cherney M.M., Withers S.G., Mahuran D.J., James M.N. (2006). Crystallographic Structure of Human Beta-Hexosaminidase A: Interpretation of Tay-Sachs Mutations and Loss of GM2 Ganglioside Hydrolysis. J. Mol. Biol..

[34] O’Dowd B.F., Cumming D.A., Gravel R.A., Mahuran D. (1988). Oligosaccharide Structure and Amino Acid Sequence of the Major Glycopeptides of Mature Human Beta-Hexosaminidase. Biochemistry.

[35] Weitz G., Proia R.L. (1992). Analysis of the Glycosylation and Phosphorylation of the Alpha-Subunit of the Lysosomal Enzyme, Beta-Hexosaminidase A, by Site-Directed Mutagenesis. J. Biol. Chem..

[36] Proia R.L., d’Azzo A., Neufeld E.F. (1984). Association of Alpha- And Beta-Subunits during the Biosynthesis of Beta-Hexosaminidase in Cultured Human Fibroblasts. J. Biol. Chem..

[37] Saftig P., Klumperman J. (2009). Lysosome Biogenesis and Lysosomal Membrane Proteins: Trafficking Meets Function. Nat. Rev. Mol. Cell Biol..

[38] Sandhoff R., Sandhoff K. (2018). Emerging Concepts of Ganglioside Metabolism. FEBS Lett..

[39] Mencarelli S., Cavalieri C., Magini A., Tancini B., Basso L., Lemansky P., Hasilik A., Li Y.T., Chigorno V., Orlacchio A. (2005). Identification of Plasma Membrane Associated Mature Beta-Hexosaminidase A, Active Towards GM2 Ganglioside, in Human Fibroblasts. FEBS Lett..

[40] Tropak M.B., Yonekawa S., Karumuthil-Melethil S., Thompson P., Wakarchuk W., Gray S.J., Walia J.S., Mark B.L., Mahuran D. (2016). Construction of a Hybrid Β-Hexosaminidase Subunit Capable of Forming Stable Homodimers that Hydrolyze GM2 Ganglioside In Vivo. Mol. Ther. Methods Clin. Dev..

[41] Lahey H.G., Webber C.J., Golebiowski D., Izzo C.M., Horn E., Taghian T., Rodriguez P., Batista A.R., Ellis L.E., Hwang M. (2020). Pronounced Therapeutic Benefit of a Single Bidirectional AAV Vector Administered Systemically in Sandhoff Mice. Mol. Ther..

[42] Maier T., Strater N., Schuette C.G., Klingenstein R., Sandhoff K., Saenger W. (2003). The X-Ray Crystal Structure of Human Beta-Hexosaminidase B Provides New Insights into Sandhoff Disease. J. Mol. Biol..

[43] Schröder M., Klima H., Nakano T., Kwon H., Quintern L.E., Gärtner S., Suzuki K., Sandhoff K. (1989). Isolation of a cDNA Encoding the Human GM2 Activator Protein. FEBS Lett..

[44] Wendeler M., Hoernschemeyer J., Hoffmann D., Kolter T., Schwarzmann G., Sandhoff K. (2004). Photoaffinity Labelling of the Human GM2-Activator Protein. Mechanistic Insight into Ganglioside GM2 Degradation. Eur. J. Biochem..

[45] Braulke T., Bonifacino J.S. (2009). Sorting of Lysosomal Proteins. Biochim. Biophys. Acta.

[46] Kleiman F.E., de Kremer R.D., de Ramirez A.O., Gravel R.A., Argaraña C.E. (1994). Sandhoff disease in Argentina: High Frequency of a Splice Site Mutation in the HEXB Gene and Correlation between Enzyme and DNA-Based Tests for Heterozygote Detection. Hum. Genet..

[47] Zampieri S., Cattarossi S., Oller Ramirez A.M., Rosano C., Lourenco C.M., Passon N., Moroni I., Uziel G., Pettinari A., Stanzial F. (2012). Sequence and Copy Number Analyses of HEXB Gene in Patients Affected by Sandhoff Disease: Functional Characterization of 9 Novel Sequence Variants. PLoS ONE.

[48] Sheth J., Datar C., Mistri M., Bhavsar R., Sheth F., Shah K. (2016). GM2 Gangliosidosis AB Variant: Novel Mutation from INDIA—A Case Report with A Review. BMC Pediatr..

[49] Martins C., Brunel-Guitton C., Lortie A., Gauvin F., Morales C.R., Mitchell G.A., Pshezhetsky A.V. (2017). Atypical Juvenile Presentation of G M2 Gangliosidosis AB in a Patient Compound-Heterozygote for c.259G ≫ T and c.164C ≫ T Mutations in the GM2A Gene. Mol. Genet. Metab. Rep..

[50] Bley A.E., Giannikopoulos O.A., Hayden D., Kubilus K., Tifft C.J., Eichler F.S. (2011). Natural History of Infantile G(M2) Gangliosidosis. Pediatrics.

[51] Maegawa G.H., Stockley T., Tropak M., Banwell B., Blaser S., Kok F., Giugliani R., Mahuran D., Clarke J.T. (2006). The Natural History of Juvenile Or Subacute GM2 Gangliosidosis: 21 New Cases and Literature Review of 134 Previously Reported. Pediatrics.

[52] Gushulak L., Hemming R., Martin D., Seyrantepe V., Pshezhetsky A., Triggs-Raine B. (2012). Hyaluronidase 1 and β-Hexosaminidase have Redundant Functions in Hyaluronan and Chondroitin Sulfate Degradation. J. Biol. Chem..

[53] Okada S., O’Brien J.S. (1969). Tay-Sachs Disease: Generalized Absence of a Beta-D-N-Acetylhexosaminidase Component. Science.

[54] Gort L., de Olano N., Macías-Vidal J., Coll M.A., Group S.G.W. (2012). GM2 Gangliosidoses in Spain: Analysis of the HEXA and HEXB Genes in 34 Tay-Sachs and 14 Sandhoff Patients. Gene.

[55] Er E., Canda E., Yazıcı H., Eraslan C., Sozmen E., Ucar S., Coker M. (2018). An Evalution of the Demographic and Clinical Characterictics of Patients with GM2 Gangliosidosis. J. Pediatr. Res..

[56] Karimzadeh P., Jafari N., Nejad Biglari H., Jabbeh Dari S., Ahmad Abadi F., Alaee M.R., Nemati H., Saket S., Tonekaboni S.H., Taghdiri M.M. (2014). GM2-Gangliosidosis (Sandhoff and Tay Sachs disease): Diagnosis and Neuroimaging Findings (An Iranian Pediatric Case Series). Iran. J. Child. Neurol..

[57] Neudorfer O., Pastores G.M., Zeng B.J., Gianutsos J., Zaroff C.M., Kolodny E.H. (2005). Late-onset Tay-Sachs Disease: Phenotypic Characterization and Genotypic Correlations in 21 Affected Patients. Genet. Med..

[58] Bellettato C., Tomanin R., Rigon L., Zanetti A., Volpi N., Scarpa M., Tomatsu S., Lavery C., Giugliani R., Harmatz P., Scarpa M., Węgrzyn G., Orii T. (2018). Glycosaminoglycans: Biosynthesis, Degradation, and Related Lysosomal Storage Disorders. Mucopolysaccharidoses Update (2 Volume Set).

[59] Sandhoff K. (2012). My Journey into the World of Sphingolipids and Sphingolipidoses. Proc. Jpn. Acad. Ser. B Phys. Biol. Sci..

[60] Adam M.P., Ardinger H.H., Pagon R.A., Wallace S.E., Bean L.J.H., Stephens K., Amemiya A. (1993). Hexosaminidase a Deficiency. GeneReviews.

[61] Leinekugel P., Michel S., Conzelmann E., Sandhoff K. (1992). Quantitative correlation between the Residual Activity of Beta-Hexosaminidase A and Arylsulfatase A and the Severity of the Resulting Lysosomal Storage disEase. Hum. Genet..

[62] Yamaguchi A., Katsuyama K., Nagahama K., Takai T., Aoki I., Yamanaka S. (2004). Possible Role of Autoantibodies in the Pathophysiology of GM2 Gangliosidoses. J. Clin. Invest..

[63] Parker H., Bigger B.W. (2019). The Role of Innate Immunity in Mucopolysaccharide Diseases. J. Neurochem..

[64] Ballabio A. (2016). The Awesome Lysosome. EMBO Mol. Med..

[65] Darios F., Stevanin G. (2020). Impairment of Lysosome Function and Autophagy in Rare Neurodegenerative Diseases. J. Mol. Biol..

[66] Yim W.W., Mizushima N. (2020). Lysosome Biology in Autophagy. Cell Discov..

[67] Setia H., Muotri A.R. (2019). Brain Organoids as a Model System for Human Neurodevelopment and Disease. Semin. Cell Dev. Biol..

[68] Allende M.L., Cook E.K., Larman B.C., Nugent A., Brady J.M., Golebiowski D., Sena-Esteves M., Tifft C.J., Proia R.L. (2018). Cerebral Organoids Derived from Sandhoff Disease-Induced Pluripotent Stem Cells Exhibit Impaired Neurodifferentiation. J. Lipid Res..

[69] Kuil L.E., López Martí A., Carreras Mascaro A., van den Bosch J.C., van den Berg P., van der Linde H.C., Schoonderwoerd K., Ruijter G.J.G., van Ham T.J. (2019). Hexb Enzyme Deficiency Leads to Lysosomal Abnormalities in Radial Glia and Microglia in Zebrafish Brain Development. Glia.

[70] Beattie R., Hippenmeyer S. (2017). Mechanisms of Radial Glia Progenitor Cell Lineage Progression. FEBS Lett..

[71] Sargeant T.J., Drage D.J., Wang S., Apostolakis A.A., Cox T.M., Cachón-González M.B. (2012). Characterization of Inducible Models of Tay-Sachs and Related Disease. PLoS Genet..

[72] Huang J.Q., Trasler J.M., Igdoura S., Michaud J., Hanal N., Gravel R.A. (1997). Apoptotic Cell Death in Mouse Models of GM2 Gangliosidosis and Observations on Human Tay-Sachs and Sandhoff Diseases. Hum. Mol. Genet..

[73] Wada R., Tifft C.J., Proia R.L. (2000). Microglial Activation Precedes Acute Neurodegeneration in Sandhoff Disease and is Suppressed by Bone Marrow Transplantation. Proc. Natl. Acad. Sci. USA.

[74] Sargeant T.J., Wang S., Bradley J., Smith N.J., Raha A.A., McNair R., Ziegler R.J., Cheng S.H., Cox T.M., Cachón-González M.B. (2011). Adeno-Associated Virus-Mediated Expression of Β-Hexosaminidase Prevents Neuronal Loss in the Sandhoff Mouse Brain. Hum. Mol. Genet..

[75] Ginzburg L., Li S.C., Li Y.T., Futerman A.H. (2008). An Exposed Carboxyl Group on Sialic Acid is Essential for Gangliosides to Inhibit Calcium Uptake via the Sarco/Endoplasmic Reticulum Ca2+-ATPase: Relevance to Gangliosidoses. J. Neurochem..

[76] Hu H., Tian M., Ding C., Yu S. (2018). The C/EBP Homologous Protein (CHOP) Transcription Factor Functions in Endoplasmic Reticulum Stress-Induced Apoptosis and Microbial Infection. Front. Immunol..

[77] Witting A., Müller P., Herrmann A., Kettenmann H., Nolte C. (2000). Phagocytic clearance of apoptotic neurons by Microglia/Brain Macrophages In Vitro: Involvement of Lectin-, Integrin-, and Phosphatidylserine-Mediated Recognition. J. Neurochem..

[78] Nonaka S., Nakanishi H. (2019). Microglial Clearance of Focal Apoptotic Synapses. Neurosci. Lett..

[79] Bradbury A.M., Peterson T.A., Gross A.L., Wells S.Z., McCurdy V.J., Wolfe K.G., Dennis J.C., Brunson B.L., Gray-Edwards H., Randle A.N. (2017). AAV-Mediated Gene Delivery Attenuates Neuroinflammation in Feline Sandhoff Disease. Neuroscience.

[80] Jeyakumar M., Thomas R., Elliot-Smith E., Smith D.A., van der Spoel A.C., d’Azzo A., Perry V.H., Butters T.D., Dwek R.A., Platt F.M. (2003). Central Nervous System Inflammation is a Hallmark of Pathogenesis in Mouse Models of Gm1 and Gm2 Gangliosidosis. Brain.

[81] Gray-Edwards H.L., Randle A.N., Maitland S.A., Benatti H.R., Hubbard S.M., Canning P.F., Vogel M.B., Brunson B.L., Hwang M., Ellis L.E. (2018). Adeno-Associated Virus Gene Therapy in a Sheep Model of Tay-Sachs Disease. Hum. Gene Ther..

[82] Ogawa Y., Furusawa E., Saitoh T., Sugimoto H., Omori T., Shimizu S., Kondo H., Yamazaki M., Sakuraba H., Oishi K. (2018). Inhibition of Astrocytic Adenosine Receptor A. Neurobiol. Dis..

[83] Ogawa Y., Sano T., Irisa M., Kodama T., Saito T., Furusawa E., Kaizu K., Yanagi Y., Tsukimura T., Togawa T. (2017). FcRγ-Dependent Immune Activation Initiates Astrogliosis During the Asymptomatic Phase of Sandhoff Disease Model Mice. Sci. Rep..

[84] Domingues H.S., Portugal C.C., Socodato R., Relvas J.B. (2016). Oligodendrocyte, Astrocyte, and Microglia Crosstalk in Myelin Development, Damage, and Repair. Front. Cell Dev. Biol..

[85] Liddelow S.A., Barres B.A. (2017). Reactive Astrocytes: Production, Function, and Therapeutic Potential. Immunity.

[86] Haberland C., Brunngraber E., Witting L., Brown B. (1973). The White Matter in G M2 Gangliosidosis. A Comparative Histopathological and Biochemical Study. Acta Neuropathol..

[87] Tavasoli A.R., Parvaneh N., Ashrafi M.R., Rezaei Z., Zschocke J., Rostami P. (2018). Clinical Presentation and Outcome in Infantile Sandhoff Disease: A Case Series of 25 Patients from Iranian Neurometabolic Bioregistry with Five Novel Mutations. Orphanet. J. Rare Dis..

[88] Boado R.J., Lu J.Z., Hui E.K., Lin H., Pardridge W.M. (2019). Bi-Functional IgG-Lysosomal Enzyme Fusion Proteins for Brain Drug Delivery. Sci. Rep..

[89] Boado R.J., Hui E.K., Lu J.Z., Pardridge W.M. (2012). Glycemic Control and Chronic Dosing of Rhesus Monkeys with a Fusion Protein of Iduronidase and a Monoclonal Antibody against the Human Insulin Receptor. Drug. Metab. Dispos..

[90] Boado R.J., Ka-Wai Hui E., Zhiqiang Lu J., Pardridge W.M. (2014). Insulin Receptor Antibody-Iduronate 2-Sulfatase Fusion Protein: Pharmacokinetics, Anti-Drug Antibody, and Safety Pharmacology in Rhesus Monkeys. Biotechnol. Bioeng..

[91] Boado R.J., Lu J.Z., Hui E.K., Pardridge W.M. (2014). Insulin Receptor Antibody-Sulfamidase Fusion Protein Penetrates the Primate Blood-Brain Barrier and Reduces Glycosoaminoglycans in Sanfilippo Type A Cells. Mol. Pharm..

[92] Boado R.J., Lu J.Z., Hui E.K., Lin H., Pardridge W.M. (2016). Insulin Receptor Antibody-α-N-Acetylglucosaminidase Fusion Protein Penetrates the Primate Blood-Brain Barrier and Reduces Glycosoaminoglycans in Sanfilippo Type B Fibroblasts. Mol. Pharm..

[93] Beard H., Luck A.J., Hassiotis S., King B., Trim P.J., Snel M.F., Hopwood J.J., Hemsley K.M. (2015). Determination of the Role of Injection Site on the Efficacy of Intra-CSF Enzyme Replacement Therapy in MPS IIIA Mice. Mol. Genet. Metab..

[94] Schulz A., Ajayi T., Specchio N., de Los Reyes E., Gissen P., Ballon D., Dyke J.P., Cahan H., Slasor P., Jacoby D. (2018). Study of Intraventricular Cerliponase Alfa for CLN2 Disease. N. Engl. J. Med..

[95] Li M. (2018). Enzyme Replacement Therapy: A Review and Its Role in Treating Lysosomal Storage Diseases. Pediatr. Ann..

[96] Garbade S.F., Zielonka M., Mechler K., Kolker S., Hoffmann G.F., Staufner C., Mengel E., Ries M. (2020). FDA Orphan Drug Designations for Lysosomal Storage Disorders—A Cross-Sectional Analysis. PLoS ONE.

[97] Johnson W.G., Desnick R.J., Long D.M., Sharp H.L., Krivit W., Brady B., Brady R.O. (1973). Intravenous Injection of Purified Hexosaminidase a into a Patient with Tay-Sachs Disease. Birth Defects Orig. Artic. Ser..

[98] Matsuoka K., Tsuji D., Aikawa S., Matsuzawa F., Sakuraba H., Itoh K. (2010). Introduction of an N-glycan Sequon into HEXA Enhances Human Beta-Hexosaminidase Cellular Uptake in a Model of Sandhoff Disease. Mol. Ther..

[99] Tsuji D., Akeboshi H., Matsuoka K., Yasuoka H., Miyasaki E., Kasahara Y., Kawashima I., Chiba Y., Jigami Y., Taki T. (2011). Highly Phosphomannosylated Enzyme Replacement Therapy for GM2 Gangliosidosis. Ann. Neurol..

[100] Matsuoka K., Tamura T., Tsuji D., Dohzono Y., Kitakaze K., Ohno K., Saito S., Sakuraba H., Itoh K. (2011). Therapeutic Potential of Intracerebroventricular Replacement of Modified Human β-Hexosaminidase B for GM2 Gangliosidosis. Mol. Ther..

[101] Espejo-Mojica A.J., Mosquera A., Rodrfguez-Lopez A., Diaz D., Beltran L., Hernandez F.L., Alméciga-Diaz C.J., Barrera L.A. (2016). Characterization of Recombinant Human Lysosomal Beta-Hexosaminidases Produced in the Methylotrophic Yeast Pichia Pastoris. Univ. Scientiarum.

[102] Espejo-Mojica A.J., Almeciga-Diaz C.J., Rodriguez A., Mosquera A., Diaz D., Beltran L., Diaz S., Pimentel N., Moreno J., Sanchez J. (2015). Human Recombinant Lysosomal Enzymes Produced in Microorganisms. Mol. Genet. Metab..

[103] Almeciga-Diaz C.J., Espejo-Mojica A., Rodriguez-López A., Losada C., Sanchez J., Ramírez A.M., Pimentel N., Beltran L.M., Diaz D., Barrera L.A. (2016). Cell Uptake Evaluation of Human Recombinant Lysosomal Enzymes Produced in Pichia Pastoris. Mol. Genet. Metab..

[104] Vu M., Li R., Baskfield A., Lu B., Farkhondeh A., Gorshkov K., Motabar O., Beers J., Chen G., Zou J. (2018). Neural Stem Cells for Disease Modeling and Evaluation of Therapeutics for Tay-Sachs Disease. Orphanet J. Rare Dis..

[105] Pulido Z., Cifuentes J., Benincore E., Garzon R., Castellanos M.C., Leal A., Cruz J.C., Almeciga-Diaz C.J., Espejo-Mojica A.J. (2020). Recombinant Hexosaminidases Conjugated to Magnetite Nanoparticles: Alternative Therapeutic Treatment Routes in GM2 Fibroblasts. Mol. Genet. Metab..

[106] Biffi A. (2017). Hematopoietic Stem Cell Gene Therapy for Storage Disease: Current and New Indications. Mol. Ther..

[107] Sawamoto K., Chen H.H., Almeciga-Diaz C.J., Mason R.W., Tomatsu S. (2018). Gene therapy for Mucopolysaccharidoses. Mol. Genet. Metab..

[108] Hatzimichael E., Tuthill M. (2010). Hematopoietic Stem Cell Transplantation. Stem. Cells Cloning.

[109] Hobbs J.R., Hugh-Jones K., Barrett A.J., Byrom N., Chambers D., Henry K., James D.C., Lucas C.F., Rogers T.R., Benson P.F. (1981). Reversal of Clinical Features of Hurler’s Disease and Biochemical Improvement after Treatment by Bone-Marrow Transplantation. Lancet.

[110] Visigalli I., Delai S., Politi L.S., Di Domenico C., Cerri F., Mrak E., D’Isa R., Ungaro D., Stok M., Sanvito F. (2010). Gene Therapy Augments the Efficacy of Hematopoietic Cell Transplantation and Fully Corrects Mucopolysaccharidosis Type I Phenotype in the Mouse Model. Blood.

[111] Gleitz H.F., Liao A.Y., Cook J.R., Rowlston S.F., Forte G.M., D’Souza Z., O’Leary C., Holley R.J., Bigger B.W. (2018). Brain-targeted Stem Cell Gene Therapy Corrects Mucopolysaccharidosis Type Ii via Multiple Mechanisms. EMBO Mol. Med..

[112] Somaraju U.R., Tadepalli K. (2017). Hematopoietic Stem Cell Transplantation for Gaucher Disease. Cochrane Database Syst. Rev..

[113] Jacobs J.F., Willemsen M.A., Groot-Loonen J.J., Wevers R.A., Hoogerbrugge P.M. (2005). Allogeneic BMT followed by Substrate Reduction Therapy in a Child With Subacute Tay-Sachs Disease. Bone Marrow Transpl..

[114] Prasad V.K., Mendizabal A., Parikh S.H., Szabolcs P., Driscoll T.A., Page K., Lakshminarayanan S., Allison J., Wood S., Semmel D. (2008). Unrelated Donor Umbilical Cord Blood Transplantation for Inherited Metabolic Disorders in 159 Pediatric Patients from a Single Center: Influence of Cellular Composition of the Graft on Transplantation Outcomes. Blood.

[115] Ornaghi F., Sala D., Tedeschi F., Maffia M.C., Bazzucchi M., Morena F., Valsecchi M., Aureli M., Martino S., Gritti A. (2020). Novel Bicistronic Lentiviral Vectors Correct Β-Hexosaminidase Deficiency in Neural and Hematopoietic Stem Cells and Progeny: Implications for In Vivo And Ex Vivo Gene Therapy of GM2 Gangliosidosis. Neurobiol. Dis..

[116] Madden K., Chabot-Richards D. (2019). HLA Testing in the Molecular Diagnostic Laboratory. Virchows Arch..

[117] Han X., Wang M., Duan S., Franco P.J., Kenty J.H., Hedrick P., Xia Y., Allen A., Ferreira L.M.R., Strominger J.L. (2019). Generation of Hypoimmunogenic Human Pluripotent Stem Cells. Proc. Natl. Acad. Sci. USA.

[118] Deuse T., Hu X., Gravina A., Wang D., Tediashvili G., De C., Thayer W.O., Wahl A., Garcia J.V., Reichenspurner H. (2019). Hypoimmunogenic Derivatives of Induced Pluripotent Stem Cells Evade Immune Rejection in Fully Immunocompetent Allogeneic Recipients. Nat. Biotechnol..

[119] Biffi A. (2016). Gene Therapy for Lysosomal Storage Disorders: A Good Start. Hum. Mol. Genet..

[120] Arakawa T., Ejima D., Kita Y., Tsumoto K. (2006). Small Molecule Pharmacological Chaperones: From Thermodynamic Stabilization to Pharmaceutical Drugs. Biochim. Biophys. Acta.

[121] Cohen F.E., Kelly J.W. (2003). Therapeutic Approaches to Protein-Misfolding Diseases. Nature.

[122] Losada Díaz J.C., Cepeda del Castillo J., Rodriguez-López E.A., Alméciga-Díaz C.J. (2020). Advances in the Development of Pharmacological Chaperones for the Mucopolysaccharidoses. Int. J. Mol. Sci..

[123] Boyd R.E., Lee G., Rybczynski P., Benjamin E.R., Khanna R., Wustman B.A., Valenzano K.J. (2013). Pharmacological Chaperones as Therapeutics for Lysosomal Storage Diseases. J. Med. Chem..

[124] Liguori L., Monticelli M., Allocca M., Hay Mele B., Lukas J., Cubellis M.V., Andreotti G. (2020). Pharmacological Chaperones: A Therapeutic Approach for Diseases Caused by Destabilizing Missense Mutations. Int. J. Mol. Sci..

[125] Cortez L., Sim V. (2014). The Therapeutic Potential of Chemical Chaperones in Protein Folding Diseases. Prion.

[126] Maegawa G.H., Tropak M., Buttner J., Stockley T., Kok F., Clarke J.T., Mahuran D.J. (2007). Pyrimethamine as a Potential Pharmacological Chaperone for Late-Onset Forms of GM2 Gangliosidosis. J. Biol. Chem..

[127] Bateman K.S., Cherney M.M., Mahuran D.J., Tropak M., James M.N. (2011). Crystal structure of β-Hexosaminidase B in Complex with Pyrimethamine, a Potential Pharmacological Chaperone. J. Med. Chem..

[128] Chiricozzi E., Niemir N., Aureli M., Magini A., Loberto N., Prinetti A., Bassi R., Polchi A., Emiliani C., Caillaud C. (2014). Chaperone Therapy for GM2 Gangliosidosis: Effects of Pyrimethamine on Β-Hexosaminidase Activity in Sandhoff Fibroblasts. Mol. Neurobiol..

[129] Clarke J.T., Mahuran D.J., Sathe S., Kolodny E.H., Rigat B.A., Raiman J.A., Tropak M.B. (2011). An Open-Label Phase I/II Clinical Trial of Pyrimethamine for the Treatment of Patients Affected with Chronic GM2 Gangliosidosis (Tay-Sachs or Sandhoff variants). Mol. Genet. Metab..

[130] Osher E., Fattal-Valevski A., Sagie L., Urshanski N., Sagiv N., Peleg L., Lerman-Sagie T., Zimran A., Elstein D., Navon R. (2015). Effect of Cyclic, Low dose Pyrimethamine Treatment in Patients With Late Onset Tay Sachs: An Open Label, Extended Pilot Study. Orphanet J. Rare Dis..

[131] Kato A., Nakagome I., Nakagawa S., Kinami K., Adachi I., Jenkinson S.F., Désiré J., Blériot Y., Nash R.J., Fleet G.W.J. (2017). In silico Analyses Of Essential Interactions of Iminosugars with the Hex A Active Site and Evaluation of their Pharmacological Chaperone Effects for Tay-Sachs Disease. Org. Biomol. Chem..

[132] Chen Y., Jian J., Hettinghouse A., Zhao X., Setchell K., Sun Y., Liu C. (2018). Progranulin Associates with Hexosaminidase A and Ameliorates GM2 ganglioside Accumulation and Lysosomal Storage in Tay-Sachs Disease. J. Mol. Med..

[133] Jian J., Tian Q.Y., Hettinghouse A., Zhao S., Liu H., Wei J., Grunig G., Zhang W., Setchell K.D.R., Sun Y. (2016). Progranulin Recruits HSP70 to β-Glucocerebrosidase and is Therapeutic against Gaucher Disease. EBioMedicine.

[134] Platt F.M., Jeyakumar M. (2008). Substrate Reduction Therapy. Acta Paediatr..

[135] Platt F.M., Neises G.R., Dwek R.A., Butters T.D. (1994). N-butyldeoxynojirimycin is a Novel Inhibitor of Glycolipid Biosynthesis. J. Biol. Chem..

[136] Pastores G.M., Barnett N.L., Kolodny E.H. (2005). An Open-Label, Noncomparative Study of Miglustat in Type I Gaucher Disease: Efficacy and Tolerability over 24 Months of Treatment. Clin. Ther..

[137] Zervas M., Somers K.L., Thrall M.A., Walkley S.U. (2001). Critical Role for Glycosphingolipids in Niemann-Pick Disease Type C. Curr. Biol..

[138] Elliot-Smith E., Speak A.O., Lloyd-Evans E., Smith D.A., van der Spoel A.C., Jeyakumar M., Butters T.D., Dwek R.A., d’Azzo A., Platt F.M. (2008). Beneficial Effects of Substrate Reduction Therapy in a Mouse Model of GM1 Gangliosidosis. Mol. Genet. Metab..

[139] Platt F.M., Jeyakumar M., Andersson U., Heare T., Dwek R.A., Butters T.D. (2003). Substrate Reduction Therapy in Mouse Models of the Glycosphingolipidoses. Philos. Trans. R. Soc. Lond. B Biol. Sci..

[140] Jeyakumar M., Butters T.D., Cortina-Borja M., Hunnam V., Proia R.L., Perry V.H., Dwek R.A., Platt F.M. (1999). Delayed Symptom Onset and Increased Life Expectancy in Sandhoff Disease Mice Treated with N-Butyldeoxynojirimycin. Proc. Natl. Acad. Sci. USA.

[141] Platt F.M., Neises G.R., Reinkensmeier G., Townsend M.J., Perry V.H., Proia R.L., Winchester B., Dwek R.A., Butters T.D. (1997). Prevention of Lysosomal Storage in Tay-Sachs Mice Treated with N-Butyldeoxynojirimycin. Science.

[142] Maegawa G.H., Banwell B.L., Blaser S., Sorge G., Toplak M., Ackerley C., Hawkins C., Hayes J., Clarke J.T. (2009). Substrate Reduction Therapy in Juvenile GM2 Gangliosidosis. Mol. Genet. Metab..

[143] Masciullo M., Santoro M., Modoni A., Ricci E., Guitton J., Tonali P., Silvestri G. (2010). Substrate Reduction Therapy with Miglustat in Chronic GM2 Gangliosidosis Type SANDHOFF: Results of a 3-Year Follow-Up. J. Inherit. Metab. Dis..

[144] Ashe K.M., Bangari D., Li L., Cabrera-Salazar M.A., Bercury S.D., Nietupski J.B., Cooper C.G., Aerts J.M., Lee E.R., Copeland D.P. (2011). Iminosugar-Based Inhibitors of Glucosylceramide Synthase Increase Brain Glycosphingolipids and Survival in a Mouse Model of Sandhoff Disease. PLoS ONE.

[145] Glass C.K., Saijo K., Winner B., Marchetto M.C., Gage F.H. (2010). Mechanisms Underlying Inflammation in Neurodegeneration. Cell.

[146] Arthur J.R., Wilson M.W., Larsen S.D., Rockwell H.E., Shayman J.A., Seyfried T.N. (2013). Ethylenedioxy-PIP2 Oxalate Reduces Ganglioside Storage in Juvenile Sandhoff Disease Mice. Neurochem. Res..

[147] Ohashi T. (2019). Gene Therapy for Lysosomal Storage Diseases And Peroxisomal Diseases. J. Hum. Genet..

[148] Schneller J.L., Lee C.M., Bao G., Venditti C.P. (2017). Genome Editing for Inborn Errors of Metabolism: Advancing towards the Clinic. BMC Med..

[149] Solovyeva V.V., Shaimardanova A.A., Chulpanova D.S., Kitaeva K.V., Chakrabarti L., Rizvanov A.A. (2018). New Approaches to Tay-Sachs Disease Therapy. Front. Physiol..

[150] Rabinowitz J., Chan Y.K., Samulski R.J. (2019). Adeno-Associated Virus (AAV) Versus Immune Response. Viruses.

[151] Mays L.E., Wilson J.M. (2011). The Complex and Evolving Story of T Cell Activation to AAV Vector-Encoded Transgene Products. Mol. Ther..

[152] Barnes C., Scheideler O., Schaffer D. (2019). Engineering the AAV Capsid to Evade Immune responses. Curr. Opin. Biotechnol..

[153] Cachón-González M.B., Wang S.Z., McNair R., Bradley J., Lunn D., Ziegler R., Cheng S.H., Cox T.M. (2012). Gene Transfer Corrects Acute GM2 Gangliosidosis—Potential Therapeutic Contribution of Perivascular Enzyme Flow. Mol. Ther..

[154] Osmon K.J., Woodley E., Thompson P., Ong K., Karumuthil-Melethil S., Keimel J.G., Mark B.L., Mahuran D., Gray S.J., Walia J.S. (2016). Systemic Gene Transfer of a Hexosaminidase Variant Using an scAAV9.47 Vector Corrects GM2 Gangliosidosis in Sandhoff Mice. Hum. Gene Ther..

[155] Jiang F., Doudna J.A. (2017). CRISPR-Cas9 Structures and Mechanisms. Annu. Rev. Biophys..

[156] Sung P. (2018). Introduction to the Thematic Minireview Series: DNA double-strand break repair and pathway choice. J. Biol. Chem..

[157] Wilson L.O.W., O’Brien A.R., Bauer D.C. (2018). The Current State and Future of CRISPR-Cas9 gRNA Design Tools. Front. Pharmacol..

[158] Zhang H.X., Zhang Y., Yin H. (2019). Genome Editing with mRNA Encoding ZFN, TALEN, and Cas9. Mol. Ther..

[159] Jinek M., Chylinski K., Fonfara I., Hauer M., Doudna J.A., Charpentier E. (2012). A programmable dual-RNA-guided DNA endonuclease in adaptive bacterial immunity. Science.

[160] Rockwell H.E., McCurdy V.J., Eaton S.C., Wilson D.U., Johnson A.K., Randle A.N., Bradbury A.M., Gray-Edwards H.L., Baker H.J., Hudson J.A. (2015). AAV-Mediated Gene Delivery in a Feline Model of Sandhoff Disease Corrects Lysosomal Storage in the Central Nervous System. ASN Neuro.

[161] Golebiowski D., van der Bom I.M.J., Kwon C.S., Miller A.D., Petrosky K., Bradbury A.M., Maitland S., Kühn A.L., Bishop N., Curran E. (2017). Direct Intracranial Injection of AAVrh8 Encoding Monkey β-N-Acetylhexosaminidase Causes Neurotoxicity in the Primate Brain. Hum. Gene Ther..

[162] Arfi A., Bourgoin C., Basso L., Emiliani C., Tancini B., Chigorno V., Li Y.T., Orlacchio A., Poenaru L., Sonnino S. (2005). Bicistronic Lentiviral Vector Corrects Beta-Hexosaminidase Deficiency in Transduced and Cross-Corrected Human Sandhoff Fibroblasts. Neurobiol. Dis..

[163] Woodley E., Osmon K.J.L., Thompson P., Richmond C., Chen Z., Gray S.J., Walia J.S. (2019). Efficacy of a Bicistronic Vector for Correction of Sandhoff Disease in a Mouse Model. Mol. Ther. Methods Clin. Dev..

[164] Ho B.X., Loh S.J.H., Chan W.K., Soh B.S. (2018). In Vivo Genome Editing as a Therapeutic Approach. Int. J. Mol. Sci..

[165] Ishii A., Kurosawa A., Saito S., Adachi N. (2014). Analysis of the Role of Homology Arms in Gene-Targeting Vectors in Human Cells. PLoS ONE.

[166] Kanca O., Zirin J., Garcia-Marques J., Knight S.M., Yang-Zhou D., Amador G., Chung H., Zuo Z., Ma L., He Y. (2019). An Efficient CRISPR-Based Strategy to Insert Small and Large Fragments of DNA Using Short Homology Arms. Elife.

[167] Anzalone A.V., Randolph P.B., Davis J.R., Sousa A.A., Koblan L.W., Levy J.M., Chen P.J., Wilson C., Newby G.A., Raguram A. (2019). Search-and-Replace Genome Editing without Double-Strand Breaks or Donor DNA. Nature.

[168] Keijzers G., Bohr V.A., Rasmussen L.J. (2015). Human exonuclease 1 (EXO1) Activity Characterization and its Function on Flap Structures. Biosci. Rep..

[169] Nami F., Basiri M., Satarian L., Curtiss C., Baharvand H., Verfaillie C. (2018). Strategies for In Vivo Genome Editing in Nondividing Cells. Trends Biotechnol..

[170] Papapetrou E.P., Schambach A. (2016). Gene Insertion into Genomic Safe Harbors for Human Gene Therapy. Mol. Ther..

[171] Ou L., Przybilla M.J., Tăbăran A.F., Overn P., O’Sullivan M.G., Jiang X., Sidhu R., Kell P.J., Ory D.S., Whitley C.B. (2020). A novel gene editing system to treat both Tay-Sachs and Sandhoff diseases. Gene Ther..

[172] Cohen J. (2019). Prime Editing Promises to be a Cut above CRISPR. Science.

[173] Santos R., Amaral O. (2019). Advances in Sphingolipidoses: CRISPR-Cas9 Editing as an Option for Modelling and Therapy. Int. J. Mol. Sci..

[174] Ledford H. (2019). Super-Precise New CRISPR Tool could Tackle a Plethora of Genetic Diseases. Nature.

[175] Sawamoto K., Stapleton M., Almeciga-Diaz C.J., Espejo-Mojica A.J., Losada J.C., Suarez D.A., Tomatsu S. (2019). Therapeutic Options for Mucopolysaccharidoses: Current and Emerging Treatments. Drugs.

[176] Hassan S., Sidransky E., Tayebi N. (2017). The Role of Epigenetics in Lysosomal Storage Disorders: Uncharted Territory. Mol. Genet. Metab..

[177] Rutten M.G.S., Rots M.G., Oosterveer M.H. (2020). Exploiting Epigenetics for the Treatment of Inborn Errors of Metabolism. J. Inherit. Metab. Dis..

